# Effectiveness of Systematic Echocardiographic Screening for Rheumatic Heart Disease in Nepalese Schoolchildren: A Cluster Randomized Clinical Trial

**DOI:** 10.1001/jamacardio.2020.7050

**Published:** 2021-01-20

**Authors:** Prahlad Karki, Surendra Uranw, Santosh Bastola, Rajan Mahato, Nikesh Raj Shrestha, Kunjang Sherpa, Sahadeb Dhungana, Ayodele Odutayo, Keshar Gurung, Naveen Pandey, Krishna Agrawal, Prashant Shah, Martina Rothenbühler, Peter Jüni, Thomas Pilgrim

**Affiliations:** 1 Department of Internal Medicine and Cardiology, B.P. Koirala Institute of Health Sciences, Dharan, Nepal; 2 Department of Cardiology, Neuro Cardio and Multispeciality Hospital, Biratnagar, Nepal; 3 Noble Medical College Teaching Hospital and Research Center, Biratnagar, Nepal; 4 Applied Health Research Centre, Li Ka Shing Knowledge Institute of St Michael’s Hospital, Department of Medicine and Institute of Health Policy, Management, and Evaluation, University of Toronto, Toronto, Ontario, Canada; 5 Department of Cardiology, Inselspital, University of Bern, Bern, Switzerland

## Abstract

**Importance:**

Echocardiographic screening allows for early detection of subclinical stages of rheumatic heart disease among children in endemic regions.

**Objective:**

To investigate the effectiveness of systematic echocardiographic screening in combination with secondary antibiotic prophylaxis on the prevalence of rheumatic heart disease.

**Design, Setting, and Participants:**

This cluster randomized clinical trial included students 9 to 16 years of age attending public and private schools in urban and rural areas of the Sunsari district in Nepal that had been randomly selected on November 17, 2012. Echocardiographic follow-up was performed between January 7, 2016, and January 3, 2019.

**Interventions:**

In the experimental group, children underwent systematic echocardiographic screening followed by secondary antibiotic prophylaxis in case they had echocardiographic evidence of latent rheumatic heart disease. In the control group, children underwent no echocardiographic screening.

**Main Outcomes and Measures:**

Prevalence of the composite of definite or borderline rheumatic heart disease according to the World Heart Federation criteria in experimental and control schools as assessed 4 years after intervention.

**Results:**

A total of 35 schools were randomized to the experimental group (n = 19) or the control group (n = 16). After a median of 4.3 years (interquartile range [IQR], 4.0-4.5 years), 17 of 19 schools in the experimental group (2648 children; median age at follow-up, 12.1 years; IQR, 10.3-12.5 years; 1308 [49.4%] male) and 15 of 16 schools in the control group (1325 children; median age at follow-up, 10.6 years; IQR, 10.0-12.5 years; 682 [51.5%] male) underwent echocardiographic follow-up. The prevalence of definite or borderline rheumatic heart disease was 10.8 per 1000 children (95% CI, 4.7-24.7) in the control group and 3.8 per 1000 children (95% CI, 1.5-9.8) in the experimental group (odds ratio, 0.34; 95% CI, 0.11-1.07; *P* = .06). The prevalence in the experimental group at baseline had been 12.9 per 1000 children (95% CI, 9.2-18.1). In the experimental group, the odds ratio of definite or borderline rheumatic heart disease at follow-up vs baseline was 0.29 (95% CI, 0.13-0.65; *P* = .008).

**Conclusions and Relevance:**

School-based echocardiographic screening in combination with secondary antibiotic prophylaxis in children with evidence of latent rheumatic heart disease may be an effective strategy to reduce the prevalence of definite or borderline rheumatic heart disease in endemic regions.

**Trial Registration:**

ClinicalTrials.gov Identifier: NCT01550068

## Key Points

**Question:**

Is echocardiographic screening of schoolchildren in regions with an endemic pattern of rheumatic heart disease followed by secondary antibiotic prophylaxis in children with evidence of subclinical rheumatic heart disease effective in reducing the prevalence of rheumatic heart disease?

**Findings:**

This cluster randomized clinical trial found a nonstatistically significant lower prevalence of definite or borderline rheumatic heart disease in schools with echocardiographic screening 4 years after intervention compared with control schools with no previous screening. An auxiliary repeated cross-sectional analysis of experimental schools found a significant reduction in the odds of definite or borderline rheumatic heart disease.

**Meaning:**

A lower prevalence of rheumatic heart disease in schools with prior echocardiographic screening warrants further study of the effectiveness of early detection and timely treatment of rheumatic heart disease in children and adolescents.

## Teaser

This cluster randomized clinical trial investigates the effect of systematic echocardiographic screening in combination with secondary antibiotic prophylaxis on the prevalence of rheumatic heart disease in schoolchildren in Nepal.

## Introduction

Rheumatic heart disease (RHD) is acquired during childhood and causes more than 10 million disability-adjusted life-years every year, accounting for most deaths from valvular heart disease worldwide.^1,2^ The burden of RHD is disproportionately prevalent in marginalized communities across sub-Saharan Africa, South Asia, and the Pacific Islands, where endemic patterns of disease prevail.^1,3^ Strategies to target RHD include secondary antibiotic prophylaxis in children with a history of acute rheumatic fever. Secondary antibiotic prophylaxis is recommended by the World Health Organization^4^ and has been associated with a reduced risk of recurrence of acute rheumatic fever and death.^5^ The effectiveness of secondary prophylaxis is, however, limited by the often oligosymptomatic course of rheumatic fever, resulting in a substantial burden of latent RHD among children and adolescents in regions with an endemic pattern of disease.^6^ Echocardiographic screening has been advocated as a strategy to allow for the early detection of latent RHD.^7^ A previous cross-sectional study^8^ using echocardiographic screening for RHD in Nepal found that 10 per 1000 children had evidence of RHD, with 4 of 5 cases being latent. Early detection of subclinical stages of RHD and timely initiation of secondary antibiotic prophylaxis for latent disease may reverse subclinical valvular lesions, prevent disease progression, and contain the reservoir for further spread.^9^ There is, however, no randomized evidence available to support this notion. Herein we describe the results of a cluster randomized trial to investigate the effectiveness of systematic echocardiographic screening for latent RHD followed by secondary antibiotic prophylaxis in children with echocardiographic evidence of valvular disease in reducing the prevalence of RHD in adolescents.

## Methods

### Setting and Design

In December 2012, we launched a school-based echocardiographic screening program in an evaluable random sample of schools in the Sunsari district of Nepal, which was planned in collaboration with the district education office.^8^ The Sunsari district is situated on the foothills of the lower Himalayan range in the eastern development region of Nepal and is part of the Outer Terai of the Koshi zone. As 1 of 75 districts in Nepal, the Sunsari district extends over an area comparable in size to New York City and has a total population of more than 760 000 inhabitants. The sampling frame comprised the total of 595 primary and secondary schools in the Sunsari district registered in 2012. Central random sampling of schools, stratified by setting (rural vs urban) and administration of the school (public vs private), was performed on November 17, 2012. Within each stratum, all schools were ordered according to a computer-generated random sequence,^10^ with eligibility of schools determined according to the random sequence. Rural to urban schools were selected in a 3:1 ratio and public to private schools in a 2:1 ratio to reflect the distribution of all children in the educational system in Nepal.^11^ The trial protocol can be found in Supplement 1. Written informed consent was obtained from school principals of all included schools. Data were not deidentified. The study was approved by the institutional review board of B.P. Koirala Institute of Health Sciences and the Nepal Health Research Council. This study followed the Consolidated Standards of Reporting Trials (CONSORT) reporting guideline.

The study was initially conceived as a prospective, single-group, interventional study of the effect of echocardiographic screening followed by antibiotic treatment (if medically indicated) on the prevalence of RHD in a locally representative random sample of schools.^8^ However, in August 2013, a random sample of control schools was additionally selected at the request of the Nepal Health Research Council based on the random sequence generated in November 2012. This random control sample was selected before completion of recruitment of schools and children for the experimental group of the study and more than 2 years before initiation of follow-up in January 2016. The sampling approach allowed for a cluster randomized comparison of experimental and control schools at follow-up. Children were eligible for the randomized cluster comparison if their age at baseline was between 5 and 12 years. Schools with at least 10 children in this age range were eligible. All children in the eligible age range were approached to undergo on-site echocardiographic examination at baseline^8^ and follow-up in experimental schools and at follow-up only in control schools. Children and their parents or primary caregivers were given the opportunity to opt out. The project was accompanied by a public awareness campaign in local news media.

### Procedures

Echocardiographic follow-up examinations were performed by 2 trained physicians (K.S. and S.D.), who were blinded to study group allocation and who were unaware of the findings of previous examinations performed in the experimental schools, using a battery-powered portable ultrasound machine (MySonoU6; Samsung Medison). Children with morphologic features or pathological valvular function suggestive of RHD underwent confirmatory examination at the B.P. Koirala Institute of Health Sciences in Dharan, Nepal. Physicians (N.P. and P.S.) performing confirmatory echocardiography were blinded to whether children attended experimental or control schools. Children with definite or borderline RHD were included in a prospective registry and recommended annual clinical and echocardiographic follow-up. All children with definite RHD at any time point throughout the study were recommended to receive secondary antibiotic prophylaxis with intramuscular benzathine benzylpenicillin (600 000 IU for those weighing <30 kg and 1 200 000 IU for those weighing ≥30 kg) once a month. Children with borderline RHD were not administered a secondary antibiotic prophylaxis. All administrations of the medication were delivered by trained study personnel. Children were considered adherent if they had received at least 2 doses of intramuscular benzathine benzylpenicillin or an alternative antibiotic in the 3 months before follow-up. Details of data collection and data management are summarized in the eMethods in Supplement 2.

### Study Outcomes

The primary outcome was the prevalence of definite or borderline RHD defined according to the World Heart Federation criteria for individuals 20 years or younger in schools enrolled into the experimental vs control group at follow-up.^12^ Definite RHD was defined by at least 2 of 4 morphologic features and pathological valvular regurgitation or stenosis or borderline disease of both the aortic and the mitral valve. Borderline RHD was defined by at least 2 morphologic features of the valvular apparatus or the presence of pathological valvular regurgitation.^12^ Secondary outcomes of the within-group comparison in the experimental group included regression from definite to borderline RHD and remission of valvular pathology. In children in the experimental group who received secondary antibiotic prophylaxis, the following safety end points were prospectively recorded: death, cerebrovascular events, hospitalizations for heart failure, and allergic reactions to penicillin.

### Statistical Analysis

Assuming an intraclass correlation coefficient of 0.001,^13^ we estimated that 34 schools with a mean of 100 children 9 to 16 years of age randomized in a 1:1 ratio would yield 87% power to detect a reduction in the primary outcome from 12 per 1000 children in the control group^8^ to 2 per 1000 children in the experimental group, 77% power to detect a reduction from 12 to 3 per 1000 children, and 64% power to detect a reduction from 12 to 4 per 1000 children after a planned follow-up of approximately 3 years at a 2-sided α = .05. The sample size calculation was not adjusted for unavailability of follow-up. To compare the marginal odds of the composite of definite or borderline RHD between the experimental and control schools, we used a random-effects, multilevel logistic regression model, including setting (rural vs urban) and school administration (public vs private) as covariates at school level and age at baseline and sex as covariates at children level. In addition, we used a random intercept to account for clustering within schools. Marginal estimates were derived with respect to the median proportions of rural and public schools, the median proportion of girls, and the median of the arithmetic mean of the age of children at follow-up across all schools of the cluster randomized comparison that were followed up. To derive prevalence estimates with 95% CIs, we transformed odds and corresponding boundaries of 95% CIs to probabilities. All children who underwent follow-up were included in the analysis according to their school’s randomization. Prespecified subgroup analyses were performed according to age at baseline (5 to ≤8 years vs >8 to 12 years), sex, and school administration (public vs private) and were accompanied by a test for interaction from the random-effects multilevel model. The subgroup analysis according to setting (rural vs urban) did not converge. To compare the marginal odds of the composite of definite or borderline RHD between baseline and follow-up in a repeated cross-sectional study of experimental schools, we also included a nested random intercept in the multilevel logistic regression model to account for clustering within children. Echocardiographic outcomes of children in experimental schools with definite RHD at the screening examination at baseline were explored using spaghetti plots, and the percentage of children with regression to borderline disease or remission was compared in children with less than 2.5 years of maximal follow-up and children with 2.5 years or more of maximal follow-up using a 2-sided Fisher exact test. Projections for the expansion of the screening program to the entire district are detailed in the eMethods in Supplement 2. All analyses were conducted in Stata software, version 15 (StataCorp LLC). All *P* values and 95% CIs are 2-sided. *P* < .05 was considered to be statistically significant.

## Results

**Figure 1.**
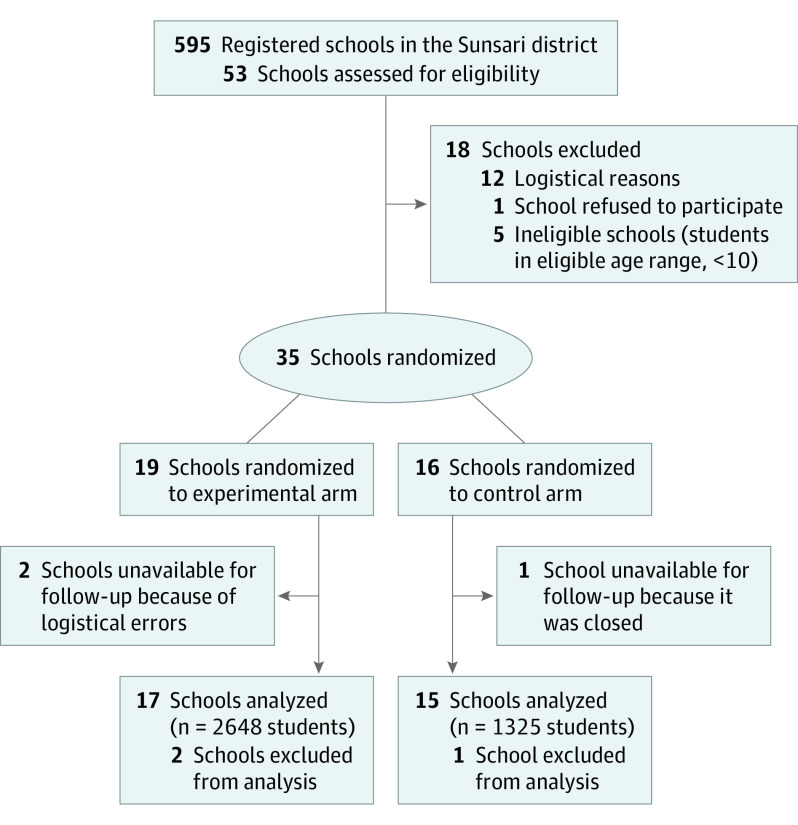
Study Flowchart According to the CONSORT Statement

Thirty-five schools were included in the study and randomized to the experimental group (n = 19) or the control group (n = 16) (Figure 1). Between December 12, 2012, and August 22, 2014, all eligible children 5 to 12 years of age from the 19 schools in the experimental group underwent echocardiographic screening at baseline, whereas no screening was performed in the 16 schools in the control group. Between January 7, 2016, and January 3, 2019, after a median interval of 4.3 years (interquartile range [IQR], 4.0-4.5 years), children aged 9 to 16 years at 17 experimental and 15 control schools underwent follow-up; 2 experimental schools were unavailable for follow-up because of a logistical error, and 1 control school had been closed after randomization. All 2648 eligible children (1308 [49.4%] male) attending the 17 experimental schools and all 1325 eligible children (682 [51.5%] male) attending the 15 control schools underwent echocardiographic ascertainment of the prespecified primary outcome. Demographic characteristics, which are summarized in the Table, were well balanced between experimental and the control schools, with exception of the median number of students per school, which was 77 (IQR, 46-249) in the experimental group and 49 (IQR, 28-101) in the control group. Six of 17 schools (35.3%) in the experimental group and 5 of 15 schools (33.3%) in the control group were private schools. The median of the arithmetic mean age of children per school at follow-up was 12.1 years (IQR, 10.3-12.5 years) in the experimental group and 10.6 years (IQR, 10.0-12.5 years) in the control group. The timeline of outcome ascertainment at follow-up in experimental and control schools is illustrated in eFigure 1 in Supplement 2.

**Table.** Demographic Characteristics of the Study Schools and Children

Characteristic	Experimental group (n = 17 schools and 2648 children)	Control group (n = 15 schools and 1325 children)
Type of school, No. (%)		
Urban area	4 (23.5)	3 (20.0)
Private	6 (35.3)	5 (33.3)
No. of children per school, median of the mean (IQR)	77 (46-249)	49 (28-101)
Age per school at baseline, median of the mean (IQR), y	8.2 (6.3-9.0)	7.5 (6.0-8.3)
Age per school at follow-up, median of the mean (IQR), y	12.1 (10.3-12.5)	10.6 (10.0-12.5)
Boys per school, No. (%) [IQR]	33 (48.9) [41.9-55.0]	23 (51.3) [42.6-56.0]

Abbreviation: IQR, interquartile range.

### Randomized Cluster Comparison

**Figure 2.**
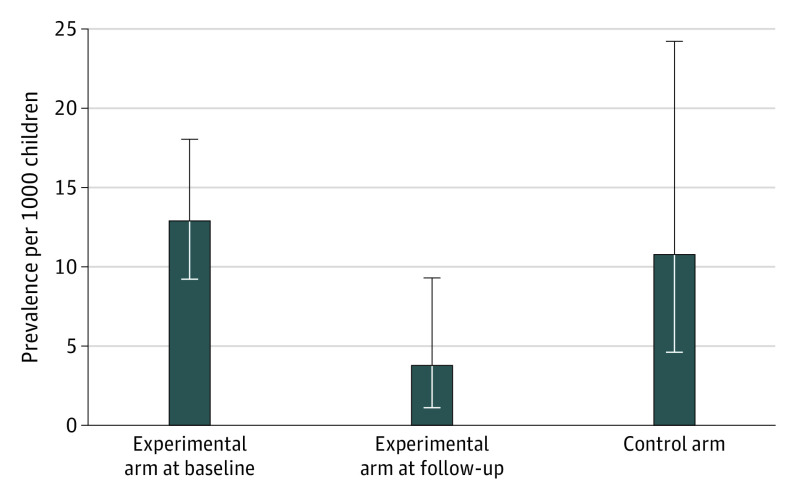
Prevalence of Definite and Borderline Rheumatic Heart Disease in Experimental Schools and Control Schools Error bars indicate 95% CIs.

**Figure 3.**
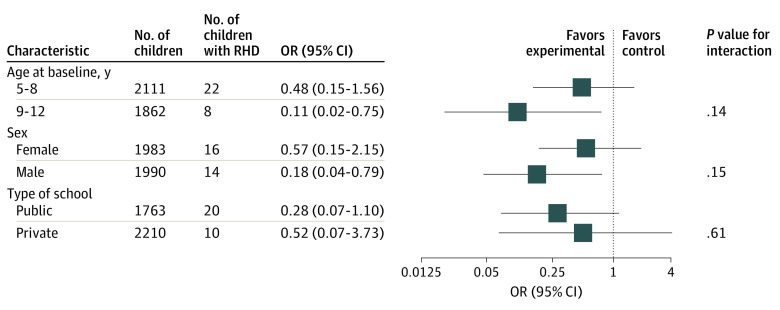
Stratified Analyses of Definite or Borderline Rheumatic Heart Disease (RHD) Across Major Subgroups All subgroup analyses were post hoc. Odds ratios (ORs) (95% CIs) are estimated using the Mantel-Cox method with 2-sided *P* values from the log-rank test. *P* values for interactions were obtained with approximate χ^2^ tests for unequal ORs in the subgroups.

At follow-up, 13 of 2648 children in experimental schools had definite (n = 2) or borderline (n = 11) RHD, and 17 of 1325 children in control schools had definite (n = 2) or borderline (n = 15) RHD. The estimated prevalence of definite or borderline RHD was 3.8 per 1000 children (95% CI, 1.5-9.8) in the experimental group and 10.8 per 1000 children (95% CI, 4.7-24.7) in the control group (odds ratio [OR], 0.34; 95% CI, 0.11-1.07; *P* = .06) (Figure 2). Intracluster correlation was 0.24 (95% CI, 0.07-0.56). In a sensitivity analysis that included only cases of definite RHD, the effect of echocardiographic screening and secondary prophylaxis in children with latent RHD was consistent with the primary end point analysis (OR, 0.49; 95%, CI 0.07-3.60; *P* = .49). In prespecified subgroup analyses, no significant interaction was found between the effect of echocardiographic screening and rural or urban setting, public or private administration of schools, age at baseline, or sex of children (Figure 3). In experimental schools, definite disease in 2 children and borderline disease in 9 children were newly detected cases, whereas 2 children with borderline disease at follow-up had been diagnosed with definite disease at the screening examination. Nine children in experimental schools who were diagnosed with definite (n = 5) or borderline disease (n = 4) at the screening examination did not have signs of RHD at follow-up. The remaining 19 children in the eligible range in experimental schools who were diagnosed with definite (n = 16) or borderline disease (n = 3) at the screening examination no longer attended experimental schools at follow-up.

### Auxiliary Repeated Cross-sectional Analysis of Experimental Schools

At the screening examination, 50 of 4181 examined children in experimental schools were diagnosed with borderline or definite RHD, with an estimated prevalence of 9.7 per 1000 (95% CI, 6.9-13.6). Among the 2940 children 9 years or older, 42 children were diagnosed with definite or borderline RHD, with an estimated prevalence of 12.9 per 1000 children (95% CI, 9.2-18.1). At the time of follow-up, 13 of 2648 children in the same age range from the same schools had definite or borderline RHD, with an estimated prevalence of 3.8 per 1000 children (95% CI, 1.5-9.8). In a comparison across time, the OR for definite or borderline RHD between follow-up and baseline examination was 0.29 (95% CI, 0.13-0.65; *P* = .008) (Figure 2).

### Follow-up of Children in Experimental Schools With Definite RHD at Screening Examination

**Figure 4.**
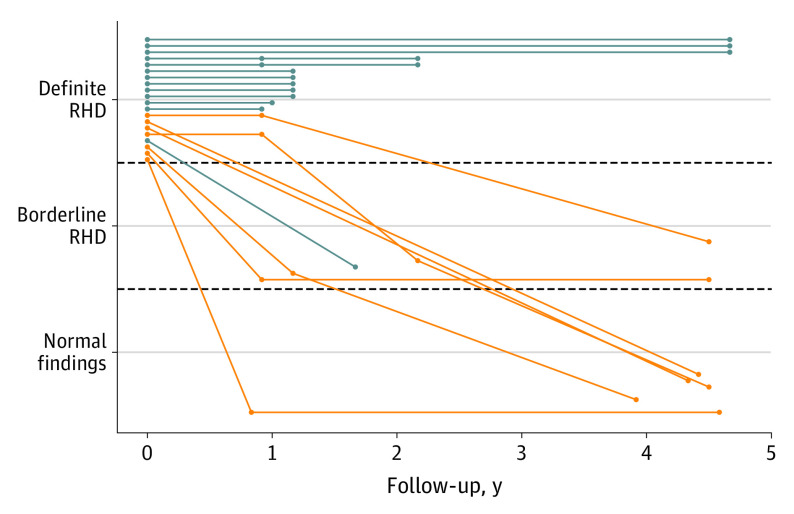
Longitudinal Data of Children in the Experimental Group With Definite Rheumatic Heart Disease (RHD) Who Underwent Follow-up Orange lines indicate children in the eligible age range who underwent follow-up in the setting of the auxiliary cross-sectional examination; blue lines, children in the eligible age range who no longer attended (the same) school.

At the screening examination, 23 children 5 to 12 years of age, who were eligible for the randomized cluster comparison, had definite RHD and were prescribed secondary prophylaxis with penicillin. Seven children could be definitively linked at follow-up at a median of 4.3 years after the screening examination (range, 3.9-4.6 years), with 5 children in remission and 2 children with regression to borderline disease. Sixteen children could not be definitively linked. Of these, 13 children had undergone additional routine echocardiographic examinations at a median follow-up of 1.2 years (range, 0.9-4.7 years), with 1 child having regression to borderline disease and 12 continuing to have echocardiographic signs of definite RHD. The remaining 3 children were unavailable for follow-up. Among the 20 children who had at least 1 follow-up, 15 (75.0%) had received at least 2 antibiotic doses in the 3 months before follow-up and were considered adherent. Figure 4 details the 20 children with any echocardiographic follow-up. Among 15 children with a maximum follow-up less than 2.5 years after the screening examination, regression or remission was documented in 4 (26.7%); among 10 children with maximum follow-up of 2.5 years or more, regression or remission was documented in 7 (70.0%) (*P* = .049). All children with regression or remission of disease had been adherent to secondary prophylaxis. eFigure 2 in Supplement 2 details the 9 children with definite RHD in the ineligible age range of 13 to 15 years at the screening examination who had undergone echocardiographic follow-up at a median of 1 year (range, 0.8-4.7 years): 2 children had a regression to borderline disease, and 7 children continued to have echocardiographic signs of definite RHD. Another 2 children with definite RHD in the ineligible age range were unavailable for follow-up. None of the children in the experimental group who began secondary prophylaxis experienced an allergic reaction to penicillin or any other safety end point.

### Projections for Studied District

We estimated that approximately 220 000 children 5 to 16 years of age lived in the Sunsari district in 2016.^11^ A 10-year strategy of 3 teams examining approximately 50 000 children annually during the first 3 years and 32 000 children annually during years 4 to 10 in a school-based screening program covering the entire district would allow the examination of 19 birth cohorts, including approximately 300 000 children at least once, with approximately 80 000 children receiving 2 examinations at an interval of 6 years. We estimated that this approach would result in the detection of 2900 children with borderline or definite RHD (95% CI, 2100-4100). The projected cost of the strategy during 10 years would be $1.4 million (eTable in Supplement 2), which corresponds to a mean of $4.67 per child examined or $483 per detected RHD case (95% CI, $341-$667).

## Discussion

In this cluster randomized clinical trial of 35 Nepalese schools, with follow-up of 32 schools with 3973 children, a lower prevalence of definite or borderline RHD in schools with echocardiographic screening 4 years prior compared with control schools with no previous screening did not reach conventional levels of statistical significance (odds ratio, 0.34; 95% CI, 0.11-1.07; *P* = .06). In an auxiliary repeated cross-sectional analysis of experimental schools, we found a significant reduction in the odds of definite or borderline RHD. The magnitudes of the reductions observed in the cluster randomized comparison and the auxiliary repeated cross-sectional analysis were similar. A 71% decrease in RHD prevalence between baseline and follow-up after implementation of an intervention with biologically plausible efficacy is corroborated by individual patient trajectories. In addition, the prevalence of RHD in the control group at follow-up was comparable to the prevalence in the experimental group at the screening examination. The consistency of these observations suggests that a strategy of echocardiographic screening and treatment of latent RHD may be effective in preventing disease progression, reversing subclinical valvular lesions, and containing the infectious reservoir. The projections made for the studied Sunsari district highlight the burden of RHD among Nepalese schoolchildren and suggest that a school-based, 10-year, systematic screening program could be implemented in the district at the modest cost of US $1.4 million.

Subclinical valvular lesions of RHD that are detected early are reversible.^14^ School-based systematic echocardiographic screening represents a pragmatic approach to detect children with early-stage disease in low-resource settings, with initiation of secondary antibiotic prophylaxis before valvular pathologies become irreversible. Focused cardiac ultrasonography by nonexperts has been associated with acceptable sensitivity and specificity for the detection of RHD and may facilitate implementation of population-based screening programs in endemic regions.^15^ When analyzing the follow-up data of the 10 children in experimental schools with definite RHD at the screening examination with a follow-up of 2.5 years or more, we found 7 children with a documented regression or remission. Among the 15 children with a shorter follow-up, only 4 had a documented regression or remission. Small prospective cohort studies found 2 of 8 children (25%) with definite RHD in Uganda^14^ and 5 of 11 children (45%) with definite RHD in Malawi^16^ with a regression or remission after approximate follow-up durations of 2 years. The randomized GwokO Adunu pa Lutino (GOAL) trial, which is currently recruiting to achieve a target sample size of 916 children, was designed to determine the effect of secondary penicillin prophylaxis on the course of latent RHD.^17^ The primary outcome of the trial will be ascertained after 2 years of follow-up. Our exploratory results suggest that the effectiveness of penicillin could increase with even longer follow-up.

Early implementation of secondary antibiotic prophylaxis represents a pragmatic approach to the silent epidemic of RHD. A comprehensive program that combines primary and secondary prevention with health education, community involvement, and epidemiologic surveillance resulted in an effective reduction of the burden of RHD in Cuba.^18^ Awareness and research funding for RHD disproportionally trails the engagement and expenditure for other diseases, such as tuberculosis and malaria, and is dwarfed by the resources spent on degenerative valvular heart disease.^19^

### Limitations

This study has several limitations. First, this was a cluster randomized clinical trial performed in a resource-constrained setting that was complicated by administrative challenges, 2 major earthquakes, and civil unrest regarding a new constitution, during which schools were closed for 4 months.^20,21,22^ These factors resulted in 2 experimental schools being unavailable for follow-up because of logistical errors and 1 control school being unavailable for follow-up because it remained closed after the civil unrest. Second, the mean follow-up of this trial was more than 1 year longer than originally planned as a direct consequence of the earthquakes and civil unrest. Third, resource constraints meant that we were only able to randomize 35 schools, with follow-up of 32 schools. In addition, the size of the schools in the control group was smaller than anticipated. Taken together, these factors meant that the power of our trial to detect a clinically meaningful reduction in definite or borderline RHD was limited. Fourth, our results cannot be generalized to children who dropped out of school early because our follow-up was restricted to children who attended school between the ages of 9 to 16 years. This limitation is important because the percentage of children out of school is considerable in Nepal and may have increased after the earthquakes and civil unrest.^11,23^ Fifth, the auxiliary repeated cross-sectional study of experimental schools was complicated by a lower-than-expected success rate in linking data between screening examination and follow-up because of errors in children-reported Nepalese names and dates of birth, variations in Western translations of names, and clustering of children-reported dates of birth. The 95% CI of the OR of the auxiliary repeated cross-sectional study is therefore conservative and would have been more narrow if a higher percentage of children had been linked in the absence of errors, variation, and clustering of identifying information. Sixth, we did not assess intrarater and interrater variability for the detection of RHD in the current study. Seventh, as per our protocol, we used adherence to antibiotic prophylaxis within the past 3 months as an approximation of adherence with antibiotic prophylaxis throughout the study period and did not use standard tools, such as records from health care professionals or patient diaries.

## Conclusion

We found evidence, albeit statistically nonsignificant, that school-based echocardiographic screening in combination with secondary antibiotic prophylaxis in children with evidence of latent RHD reduces the prevalence of definite or borderline RHD in endemic regions. A lower prevalence of RHD in schools with prior echocardiographic screening compared with control schools and a significant reduction in the prevalence of RHD in experimental schools between baseline and follow-up warrant further study of the effectiveness of early detection and timely treatment of RHD in children and adolescents.

## Notes

Supplement 1. Trial Protocol

Supplement 2. **eMethods.** Supplemental Methods **eTable.** Estimated Costs for Screening of All Schools in the Sunsari District **eFigure 1.** Timeline of Echocardiographic Follow-up in Experimental Schools (Red) and Control Schools (Blue) **eFigure 2.** Spaghetti Plot Displaying Longitudinal Data of Children in the Experimental Arm With Definite Rheumatic Heart Disease Who Were in the Ineligible Age Range for Inclusion Into the Cluster Randomized Comparison (Age >12 Years at Baseline) and Underwent Echocardiographic Follow-up Outside the Auxiliary Cross-sectional Screening **eReferences**

Supplement 3. Data sharing statement
