# Supplementary material for: Effectiveness of Systematic Echocardiographic Screening for Rheumatic Heart Disease in Nepalese Schoolchildren: A Cluster Randomized Clinical Trial
Source: JAMA Cardiol. 2021 Jan 20;6(4):1–7. doi: 10.1001/jamacardio.2020.7050 (PMC7818193; doi:10.1001/jamacardio.2020.7050)
Supplement: Supplement 1. — Trial Protocol [file jamacardiol-e207050-s001.pdf]

Research Proposal Approval Format

**Research Title:**<br>**Population-based Study of Rheumatic Heart Disease Prevalence and Cardiovascular Outcomes among Schoolchildren in Nepal**

Nepal Health Research Council (NHRC)

P.O. Box: 7626, Ramshah Path, Kathmandu, Nepal

Tel: +977-1-4254220, 4227460, Fax: +977-1-4262469

E-mail: nhrc@nhrc.org.np, Website: http://www.nhrc.org.np

For Official Use Only

(Please see the check list before Registration of the application form)

Registration No.:
Registration Date:
Approved Date:
Name of PI:
Total Budget of the Project:
NHRC Processing Fee:
Research Site:
Tentative Date of Initiating the Project:
Duration of the Research Project:
Name of Internal Reviewer:
Name of External Reviewer:
Signature & Seal of NHRC:

Part – I

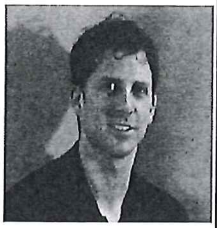

Administrative Information

1. Research Title: **Population-based Study of Rheumatic Heart Disease Prevalence and Cardiovascular Outcomes among Schoolchildren in Nepal**

2. Name and Title of Principal Investigator responsible for the proposed research:

Pilgrim	Michael	Thomas
Last (Surname)	Middle (if any)	First name

Nationality: Swiss

Citizenship Number with district name from where it was obtained (only for Nepali)

Passport Number (only for non Nepali citizen): F0265380

Signature: Th. Pilgrim Date: Oct 26 2012

Postal Address: Freiburgstrasse 10, CH-3010 Bern, Switzerland

Telephone No.: +41 31 632 21 11

Mobile No.: +41 76 548 44 11

Fax No.: +41 31 632 47 70

e-mail: thomas.pilgrim@insel.ch

Alternate e-mail: pilgrimthomas@hotmail.com

3. Full name of the Institution associated with the Principal Investigator (if applicable) : Swiss Cardiovascular Center, Bern University Hospital, Switzerland

Designation: Attending Physician

Postal Address (if different from the address given above):

Telephone No.:

Fax No.:

e-mail:

Website:

4. Declaration of the head of the Institution (if applicable)

   If the proposed research is approved, we will allow him/her to conduct the research in this institution.

   Signature: Date:

Gupta	Kumar	Arvind
Last (Surname)	Middle (if any)	First name

Designation: Hospital Director

Name of the Institution B. P. Koirala Institute of Health Sciences

Contact/Postal Address: Dharan

Telephone No.: 025-525555

Fax No.:

Institutional e-mail:

Website: www.bpkihs.edu.np

5. Name and Title of Co-investigators responsible for the proposed research (Use the similar format if more than one):

Passport size photograph

Shrestha	Raj	Nikesh
Last (Surname)	Middle (if any)	First name

Nationality: Nepali

Citizenship Number with district name from where it was obtained (only for Nepali)

Passport Number (only for non Nepali citizen):

Affiliated Institution (if applicable): BPKIHS

Designation: Associate Professor

Signature: Date:

Postal Address (if different from the address given above):

Telephone No.:

Fax No.:

e-mail:

6. Name and Title of Co-investigators responsible for the proposed research (Use the similar format if more than one):

Passport size photograph

Agrawaal	Kumar	Krishna
Last (Surname)	Middle (if any)	First name

Nationality: Nepali

Citizenship Number with district name from where it was obtained (only for Nepali) 55071 NawalParasi

Passport Number (only for non Nepali citizen):

Affiliated Institution (if applicable): BPKIHS

Designation: Senior Resident

Signature: Date:

Postal Address (if different from the address given above):

Telephone No.:

Fax No.:

e-mail: agrawalkris@gmail.com

7. Name and Title of Co-investigators responsible for the proposed research (Use the similar format if more than one):

Last (Surname)	Middle (if any)	First name
Karki		Prahlad

Passport size photograph

Nationality: Nepali

Citizenship Number with district name from where it was obtained (only for Nepali)

Passport Number (only for non Nepali citizen):

Affiliated Institution (if applicable):

Designation: Professor and Head

Signature: Date:

Postal Address (if different from the address given above):

Telephone No.:

Fax No.:

e-mail:

(Use additional sheet if necessary)

8. List the name(s) and institutional affiliation to the researcher(s) (other than co-investigator) to assist your project in Nepal and abroad (if any)

	Name	*Institution and Address*
(a)	B.P. Koirala Institute of Helath Sciences	
(b)	Swiss Cardiovascular Center, Bern	

(Use additional sheet if necessary)

9. List the name(s) of Nepali researcher(s) (other than co-investigator) or Nepalese Institution/hospital/NGO(s) etc. from whom you may seek co-operation (if any)

(a)

(b) [ ]

(Use additional sheet if necessary)

8. List major equipment(s) in relation to your research project you plan to bring/import to Nepal (If applicable)

(a) [ ]

(b) [ ]

(Use additional sheet if necessary)

8.1 List details of all specimen(s) (if any) that you may transport from Nepal in relation to your research.

(a) [ ]

(b) [ ]

(c) [ ]

(d) [ ]

8.2 Country of Destination: [ ]

Name of Institution: [ ]

8.3 Mode of Transportation of Specimen [ ]

8.4 How will you ensure duplicate specimens remain in the country?

[ ]

(If necessary use additional sheet)

[ ]

9. Is this research part of your Thesis?

Yes [ ]　　No [x]

If yes,

For what degree and in which subject? [ ]

From which university? [ ]

From which country?

Part – II

Financial Information

10.Research Title:

Protocol for a Population-based Study of Rheumatic Heart Disease Prevalence and Cardiovascular Outcomes among Schoolchildren in Nepal

11.Name of the funding organization:

Fondation de la Tour, Meyrin-Geneva, Switzerland

Contact information of funding organization or agency:

Postal Address: Av-Maillard 1, CH-1217 Meyrin-Geneva, Switzerland

Telephone No.:

Fax No.:

e-mail: purban@latour.ch

Contact person at the funding organization or agency:

Last (Surname)	Middle (if any)	First name
Urban		Philip

Designation: President of the Foundation

Total amount of funds (in NRs / US $) allocated for the proposed research project: 9885 US $

Itemized budget (in detail) and justify the resources required for the proposed research work (*use additional sheet*)

Transportation Charges: 1070 US$

OPD Ticket charge: 615 US$

Logistics: 800 US$

Salary: 6200 US$

Miscellaneous: 1200 US$

Part – III

Research Proposal Description

12.Research Title:

Protocol for a Population-based Study of Rheumatic Heart Disease Prevalence and Cardiovascular Outcomes among Schoolchildren in Nepal

13.Proposal Summary (maximum 500 words):

Introduction: Rheumatic heart disease (RHD) remains a major contributor to morbidity and mortality in developing countries. The reported prevalence rates of RHD are highly variable and mainly attributable to differences in the sensitivity of either clinical screening to detect advanced heart disease, or echocardiographic evaluation where disease is diagnosed earlier across a continuous spectrum. The clinical significance of diagnosis of subclinical RHD by echocardiographic screening and early implementation of secondary prevention has not been clearly established.

Methods and Analysis: We designed a cross-sectional survey to determine the prevalence of RHD in children from private and and public schools between the age of 5 and 15 years in urban and rural areas of Eastern Nepal using both cardiac auscultation and echocardiographic evaluation. Children with RHD will be treated with secondary prevention and enrolled in a prospective cohort study. We will compare the prevalence rates by cardiac auscultation and echocardiography, determine risk factors associated with diagnosis and progression of RHD, investigate social and economic barriers for receiving adequate cardiac care, and assess clinical outcomes with regular medical surveillance as a function of stage of disease at the time of diagnosis. Prospective clinical studies investigating the impact of secondary prevention for

subclinical RHD on long-term clinical outcome will be of central relevance for future health resource utilization in developing countries.

Ethics and Dissemination: The study was considered ethically uncritical and was given an exempt status by the ethics committee at University of Bern, Switzerland. The study has been registered with 'www.ClinicalTrials.gov' (NCT01550068). The study findings will be reported in peer-reviewed publications.

14. Introduction:

14.1 Background of Study (maximum 500 words):

Rheumatic fever complicated by rheumatic heart disease (RHD) remains a major contributor to morbidity and premature death among the socio-economically underprivileged working age population of developing countries. RHD results from an autoimmune response due to molecular mimicry between the M-protein on the group A β-hemolytic streptococci cell membrane and cardiac myosin eventually leading to valvular damage. High prevalence rates of acute rheumatic fever (ARF) and RHD have been reported from different geographic regions around the world such as Southeast Asia, the Western Pacific and Africa that share demographic characteristics determined by poverty and limited access to health care resources. The burden of RHD is likely to escalate in these countries due to increasing urbanization and overcrowding.

Prevalence rates of RHD from screening studies in Southeast Asian countries range from 0.7 to 22 per 1000 children using traditional cardiac auscultation, and from 20 to 51 per 1000 children using echocardiography. A considerable variation in prevalence rates reflects the substantially higher sensitivity of echocardiographic screening as compared to cardiac auscultation due to diagnosis across a continuous spectrum of disease as opposed to presence or absence of a heart murmur using cardiac auscultation. Echocardiographic screening for RHD has been recommended by the World Health Organization (WHO) in high prevalence regions, and the recently released criteria for echocardiographic diagnosis of RHD by the World Heart Federation (WHF) warrant consistent reporting and facilitate the evaluation of progression of minor echocardiographic lesions over time. The diagnosis of RHD at an earlier, clinically silent stage by the detection of morphological and functional valvular lesions without a corresponding heart murmur challenges our current concept of prevention and treatment.

Medical management of ARF and RHD largely depends upon preventive measures comprising reduction of overcrowding, prompt antibiotic treatment of streptococcal pharyngitis, and secondary prevention achieved by regular oral or intramuscular administration of penicillin continued until early adulthood among children with a documented history of ARF or evidence of RHD. Even though preventive measures with penicillin are inexpensive and efficient, this strategy is difficult to effectuate in developing countries with limited access to health care resources. Enrollment of patients with ARF and RHD in a

registry with close follow-up has been demonstrated to reduce the cardiovascular sequelae associated with disease progression.

14.2 Statement of the Problem and Rationale / Justification (maximum 500 words)

Effective measures to reduce the global burden of RHD represent an ongoing challenge involving reduction in overcrowding, improving hygiene, increasing public awareness and facilitating access to health care. In the absence of fundamental socio-economic changes improving primordial prevention, systematic screening for RHD based on public and private education represents the most comprehensive approach and aims at a reduction of the late complications of RHD by early implementation of secondary prevention. Current research has been predominantly focused on assessing prevalence rates using passive survey systems without subsequent enrolment in registries or offering longitudinal follow-up. In order to assess the determinants of disease and its progression along with short- and long-term clinical outcomes, we plan to include all cases of RHD in a cohort study to be treated according to their disease stage at diagnosis and followed up for at least 5 years.

14.3 Conceptual framework

The screening part of the study will be conducted for a period of 12 months. Governmental and non-governmental schools from rural and urban areas in the Sunsari district will be selected for screening in agreement with the district education office. All schoolchildren 5 to 15 years of age will be eligible for screening if parents do not withdraw consent for screening. A questionnaire designed to assess demographic characteristics and socio-economic parameters will be completed for all children.

Following a short physical examination, a focused screening echocardiography will be performed to detect rheumatic valvular lesions according to the definitions provided by the World Heart Federation. Parents of children with evidence of RHD will be contacted by the physicians and oriented about the findings. The children and parents will subsequently be invited for a thorough examination at B.P. Koirala Institute of Health Sciences. If rheumatic heart disease is confirmed in a repeat echocardiography, secondary antibiotic prevention with regular intramuscular penicillin injections will be recommended. Collaboration with family physicians and pediatricians will be central to improve long-term adherence to secondary antibiotic prevention for silent rheumatic heart disease. After informed consent for inclusion into a prospective registry, follow-up data of children with borderline or definite RHD will be collected during a dedicated follow-up visit after six months, at one year and yearly thereafter up to five years.

14.4 Research Objectives / purpose / aim of the study:

General

The objective of this study is to investigate the prevalence rate of definite and borderline RHD among children in Eastern Nepal, and to assess long-term clinical outcome of children undergoing secondary antibiotic prevention for RHD.

Specific

More specifically, we aim to (a) compare the prevalence rates by cardiac auscultation and echocardiography, (b) determine risk factors associated with diagnosis and progression of RHD, (c) investigate social and economic barriers for receiving adequate cardiac care, and (d) assess clinical outcomes as a function of stage of disease at the time of diagnosis with regular medical surveillance.

15. Research Design and Methodology

Research Method

Qualitative ☐ Quantitative ☐ Combined ☑

Study Variables:

Primary Endpoints Observational Survey:

- Borderline or Definite Rheumatic Heart Disease as defined by the World Heart Federation (WHF)
- Possible, Probable, and Definitive Rheumatic Heart Disease According to the World Health Organization (WHO)

Primary Endpoints Longitudinal Cohort Study:

- Death
- Stroke
- Endocarditis
- Recurrent rheumatic fever
- Hospitalization for Congestive Heart Failure
- Surgery for Valvular Heart Disease
- Mitral Balloon Valvuloplasty for rheumatic mitral stenosis

Type of Study (Specify):

Cross-sectional Survey (Part 1); Longitudinal Cohort Study (Part 2)

Cross-Sectional Survey (Part 1)

We will perform clinical and echocardiographic screening of children aged 5-15 years from public and private schools in urban and rural areas in Eastern Nepal. A multistage sampling procedure will be used to select the study sample. In order to ensure a representative target population, the location and administration of the schools will be used as a surrogate to reflect the socio-economic demographic distribution of the population in Eastern Nepal. Since approximately eighty percent of the population in Nepal lives in rural areas, we will include three rural and one urban area in Eastern Nepal, and enroll one third of the patients from the urban area from private schools.

Prospective Cohort Study (Part 2)

All children with documented history of ARF and/or echocardiographic evidence of RHD will be re-examined in regular time intervals, at six months, at one year, and yearly thereafter up to at least 5 years, in the context of a prospective cohort study. Both children and their primary caregivers will be educated in order to ensure compliance with secondary prevention and regular follow-up. A standardized questionnaire will address clinical symptoms, compliance to treatment, and assess pre-specified clinical

endpoints. Echocardiographic follow-up will be performed yearly up to five years at B.P. Koirala Institute of Health Sciences (BPKIHS).

Study Site and Its Justification:

The prevalence rate of RHD is high throughout Nepal. The Sunsari district situated on the foothills of the Lower Himalayan Range in Eastern Nepal involves 52 villages with a total population of around 630,000 inhabitants. Dharan is the largest city in the Sunsari district and the third largest city in the country. BPKIHS is one of the large teaching hospitals in Nepal and will be the center of reference for echocardiographic confirmation and echocardiographic follow-up of the schoolchildren.

Study Population (Specify):

For the cross-sectional survey, all parents of the schoolchildren will be informed by a letter distributed to the children outlining the project details and indicating a contact address for queries. Since close to half of the adult population in Nepal is illiterate, focus group discussions with the health care providers, school principals, local health care workers and parents will be offered to understand and establish initiatives to win the confidence of the communities. A written informed consent form of the principal of each of the selected schools will be obtained. Schoolchildren of parents that do not actively withdraw consent for screening will be examined. Inclusion criteria for the observational survey will be as follows: age 5-15 years (1), written informed consent for participation in the screening study by the school principal (2), and passive consent from the parent/primary caregiver of the children (3). Given the observational design of the study no formal exclusion criteria apply. Children will be enrolled in the prospective registry in the presence of a documented history of ARF or echocardiographic evidence of definite or borderline RHD, and written informed consent given by the children and/or their parents/primary caregivers.

Study Unit:

Governmental and nongovernmental schools in urban and rural areas of the Sunsari district

Sampling Methods / Techniques (Specify):

Cluster Randomization of Schools. In order to reflect the population of Eastern Nepal, 75% of the schools will be in rural areas, and 25% in urban areas.

Sample size (with justification):

Sample size calculations were based on reported prevalence rates of RHD using cardiac auscultation in schoolchildren in Southeast Asian countries. [1-8] We calculated a sample size of 9500 schoolchildren between the ages of 5 to 15 years with a type I error of 0.05 and a power of 90% for an expected diagnosis rate of 2 per 1000. The lower end of the prevalence estimates was chosen in order to obtain a sufficient sample size in the subsequent cohort study, and to be able to provide optimal treatment to a maximum number of affected children. We will include one urban and three rural areas from the different parts of the target area (Sunsari district), thus including 2,500 participants in each area. One third of the patients from the urban area will be enrolled from private schools.

1. Marijon E, Ou P, Celermajer DS, et al. Prevalence of rheumatic heart disease deteceted by echocardiographic screening. N Engl J Med 2007;357:470-6.
2. Bhaya M, Panwar S, Beniwal R, et al. High prevalence of rheumatic heart disease detected by echocardiography in school children. Echocardiography 2009;27:448-453.
3. Saxena A, Ramakrishnan S, Roy A, et al. Prevalence and outcome of subclinical rheumatic heart disease in India: The RHEUMATIC (Rheumatic Heart Echo Utilization and Monitoring Actuarial Trends in Indian Children) study. Heart 2011;97:2018-2022.
4. Sadiq M, Islam K, Abid R, et al. Prevalence of rheumatic heart disease in school children in urban Lahore. Heart 2009;95:353-357.
5. Bahadur KC, Sharma D, Shrestha MP, et al. Prevalence of rheumatic and congenital heart disease in schoolchildren of Kathmandu valley in Nepal. Indian Heart J 2003;5(6):615-8.
6. Jose VJ, Gomathi M. Declining prevalence of rheumatic heart disease in rural schoolchildren in India: 2001-2002. Indian Heart J 2003;55(2):158-60.
7. Ahmed J, Mostafa Zaman M, Monzur Hassan MM. Prevalence of rheumatic fever and rheumatic heart disease in rural Bangladesh. Trop Doct 2005;35(3):160-1.
8. Periwal KL, Gupta BK, Panwar RB, et al. Prevalence of rheumatic heart disease in school children in Bikaner: an echocardiographic study. J Assoc Physicians India 2006;54:279-82.

Data Collection Technique / Methods (Specify):

Data acquisition will be organized in a three-staged process by study nurses and physicians. Data on social background and past medical history will be acquired in a standardized interview on the basis of a questionnaire. Demographic variables such as age, household characteristics, and socio-economic indicators will be recorded along with a short medical history followed by physical examination documenting height, weight, and potential clinical signs of ARF. Study nurses will question the children about demographic characteristics, fill in the questionnaire, and measure height and weight. A first physician will complete the medical history and execute physical examination including cardiac auscultation. A second independent physician will perform screening echocardiography to document morphologic and/or functional valvular lesions consistent with RHD. Follow-up data will be collected during dedicated clinical visits at BPKIHS at six months, one year, and yearly thereafter up to five years. In every visit, clinical symptoms will be assessed and an echocardiographic follow-up examination will be performed.

Data Collection Tools: (please attached in annex)

Case Report Forms for the Observational Survey and the longitudinal cohort study will be provided in the annex.

Pre-testing the Data Collection Tools (if applicable):

A pilot study has been performed in order to test the data collection tools:

Objectives: To evaluate a protocol for a population-based program targeting prevention of rheumatic heart disease (RHD) progression by early echocardiographic diagnosis of valvular lesions and timely implementation of secondary prevention.

Design: Observational survey with a subsequent prospective cohort study.

Setting: Private boarding school in the urban area of the Sunsari district situated on the foothills of the Lower Himalayan Range in Eastern Nepal.

Participants: Fifty-four unselected school going children 5-15 years of age, 24 girls and 30 boys.

Primary outcome measure: Logistic feasibility of a large-scale population-based screening study using the echocardiographic criteria formulated by the World Heart Federation, with longitudinal follow-up of children with definite or borderline RHD in a prospective cohort study.

Results: Standardized interview, physical examination and screening echocardiography were performed in a three-staged process and took approximately 6 minutes per child. Socio-economic status was assessed using surrogate markers such as the occupation of the primary caregiver, numbers of rooms at home, car, television, cell phone and internet connection. Physical examination was focused on cardiac auscultation and signs of acute rheumatic fever and targeted echocardiography was performed by an independent examiner without knowledge of the clinical findings. Two children with evidence of borderline RHD were re-examined at B.P. Koirala institute of Health Sciences and the indication for secondary antibiotic prevention was discussed with the parents and the children. At six months of follow-up, echocardiographic findings were stable in both children. Implementation of secondary antibiotic prevention was challenged by impaired awareness of subclinical RHD among parents and inadequate cooperation with family physicians.

Conclusions: This pilot study shows that the methods outlined in the protocol can be translated into a large-scale population-based study. We learned that education and collaboration with teachers, parents and family physicians/pediatricians will be of key importance in order to establish a sustainable program.

Trial registration: ClinicalTrials.gov Identifier: NCT01550068

Shrestha NR, Kalesan B, Karki P, Sherpa K, Basnet A, Urban P, Pilgrim T. Rheumatic heart disease: pilot study for a population-based evaluation of prevalence and cardiovascular outcomes among schoolchildren in Nepal. BMJ Open. 2012;2(5).

Validity and Reliability of the Study Tools:

Echocardiography is the gold standard to detect clinically silent RHD. Criteria for diagnosis of RHD have been recently published by the World Heart Federation:

Echocardiographic diagnosis will classify RHD according to the WHF criteria for individuals aged ≤20 years into definite and borderline. Definite RHD is further subdivided into four subcategories. Subcategory A is pathological mitral regurgitation and at least two morphological features of RHD of the mitral valve, subcategory B is the presence of mitral stenosis with a mean gradient of ≥4 mmHg. Subcategory C is defined by pathological aortic regurgitation in combination with at least two morphological features of RHD of the aortic valve, and subcategory D is determined by borderline disease of both the aortic valve and the mitral valve. Borderline RHD is subdivided into three subcategories. Subcategory A is the presence of at least two morphological features of RHD of the mitral valve without pathological mitral regurgitation or mitral stenosis, subcategories B and C are determined by pathological mitral regurgitation, or pathological aortic regurgitation, respectively. Physiological mitral regurgitation (A), physiological aortic regurgitation (B), and an isolated morphological feature of RHD of the mitral or aortic valve (i.e. valvular thickening) without any associated pathological stenosis or regurgitation (C and D, respectively) will be classified as normal echocardiographic findings.

Potential Biases (if applicable):

Even though the population attributable risk of RHD is expected to be high, false positive screening results may occur. In order to reduce false positive findings all echocardiographic clips with borderline or definite RHD will be assessed from two independent cardiologists. Moreover, screening in a large population of schoolchildren might yield in exceptional cases important incidental clinical findings unrelated to RHD but yet relevant for future prognosis (i.e. bicuspid aortic valve, atrial septal defect). Parents will be informed about such findings and advised on how to proceed for best medical management. If the parents or care-takers give their consent, the children will be invited for follow-up at BPKIHS for further work-up. First medical contact due to incidental findings relevant for future prognosis will be reimbursed.

Limitation of the Study:

Since access to education is a marker of socio-economic status, restriction of screening to school-going children is subjected to selection bias likely to underestimate the real disease burden related to RHD in Eastern Nepal.

16.Plan for Supervision and Monitoring:

The study will be centrally monitored by the clinical trials unit at University of Bern, Switzerland.

17.Plan for Data Management and Analysis:

In the cross-sectional study, age and sex specific prevalence rates for RHD will be calculated by socio-demographic covariates. The design effect of the cluster sampling strategy will then be calculated using the variations of prevalence rates among the different clusters. A multiplication factor equal to the square root of the design effect will be used to construct the 95% confidence intervals (CIs) for the prevalence estimates to avoid erroneously narrow confidence intervals (CI). Prevalence rates with 95% CI will be calculated separately for the two screening methods. Multivariable analysis will be performed for assessing the socio-economic factors for RHD. Among children with RHD, the socio-economic barriers to receive adequate medical care will be assessed by using a multivariate logistic regression model. Furthermore, the association of RHD with age, gender, socio-economic status, and urban–rural residence will be evaluated with univariate and multivariate analyses.

Cohort baseline characteristics and procedural variables will be presented as counts and percentages for dichotomous variables and as mean and standard deviation (SD) for continuous variables. Factor analysis of the scales in QOL will be performed using appropriate rotations and index scores of the constructs will be computed if not more than 25% of the items are missing. Adherence to treatment will be measured as a dichotomous variable and quality of life (QOL) scores as mean (SD) / median (interquartile range) will be presented for each follow up time interval. We will present and compare the baseline and procedural characteristics by the stages of disease progression at baseline using chi-square tests or analyses of variance. Comparisons of the baseline characteristics of the study subjects among the disease stages at baseline will be performed using linear or logistic regression. For the specific clinical endpoints, compliance and QOL univariable and multivariable Cox proportional hazard regression models will be used to calculate hazard ratios with 95% CI among the stages of disease at baseline. We will construct Kaplan–Meier curves for the time to the development of clinical endpoints by stage of disease at baseline, treating death as a competing risk.

18. Expected Outcome of the Research:

More than 80% of the children younger than 15 years of age grow up in regions of the world where rheumatic heart disease is endemic. The results of the present study may have a far-reaching impact on health resource utilization in developing and emerging countries with endemic rheumatic heart disease where it causes 250,000 deaths every year and affects more than 15 million people. The relatively novel entity of clinically silent rheumatic heart disease introduced by the increased sensitivity of echocardiography screening challenges our current concept of secondary prevention. Diagnosis of early stages of valvular lesions due to rheumatic heart disease in school going children and timely implementation of secondary antibiotic prevention may prevent disease progression leading to morbidity and mortality.

19. Plan for Dissemination of Research Results:

The study protocol has been was registered with 'www.ClinicalTrials.gov' (NCT01550068) and was published in BMJ Open (Pilgrim T, Kalesan B, Karki P, et al. Protocol for a population-based study of rheumatic heart disease prevalence and cardiovascular outcomes among schoolchildren in Nepal. BMJ Open 2012;2(3). pii: e001320.) The study findings will be reported in peer-reviewed publications.

20. Plan for Utilization of the Research Findings (optional):

The study addresses a subject of global dimension. More than 80% of the children younger than 15 years of age grow up in regions of the world where rheumatic heart disease is endemic. The results of the present study have an important impact on health resource utilization in Nepal.

The relatively novel entity of clinically silent rheumatic heart disease introduced by the increased sensitivity of echocardiography screening challenges our current concept of secondary prevention. If the study demonstrates that secondary prevention for silent rheumatic heart disease minimizes progression of disease to adverse clinical outcome, this finding will reinforce the call for routine echocardiographic screening for rheumatic heart disease and require re-consideration of the current guidelines for secondary antibiotic prevention.

How is the research project going to strengthen the research capability of the host institution: Nepali Researcher (if submitted from abroad):

The scientific exchange will serve as a platform for mutual learning. The local research coordinators will be subjected to a training in good clinical practice and will be introduced into database management and basic statistical analyses. Clinical fellows from B.P. Koirala Institute of Health Sciences participating in the screening process will be trained to perform screening echocardiography examinations for rheumatic heart disease.

As an institution, BPKISH will be involved in an international network of rheumatic heart disease research and closely collaborate with the Clinical Trials Unit at Bern University Hospital in Switzerland.

21. Work Plan *(should include duration of study, tentative date of starting the project and work schedule / Gantt chart):*

Date	Milestone	Responsibility
Approval Institutional review board, ethics committee; registration with www.clinicaltrials.gov	February 2012	Cardiology, Bern<br>Cardiology, BPKIHS
Publication of study protocol	May 2012	
Pilot Study for phase I and phase II	February to August 2012	Cardiology, Bern<br>Cardiology, BPKIHS
Publication of pilot study	September 2012	
Cluster randomization of schools, submission to district education office	Septem ber 2012	Cardiology, Bern<br>Cardiology, BPKIHS
Orientation of school principals	September 2012	Cardiology, BPKIHS
Orientation of parents about observational survey	September 2012	Cardiology, Bern<br>Cardiology, BPKIHS
Installation of REDCap electronic data capture tools	September 2012	ISPM, Bern
Cross-sectional survey	September 2012 to August 2013	Cardiology, BPKIHS
Publication of cross-sectional survey	September 2013	

Longitudinal cohort study	September 2013 to August 2016	Cardiology, BPKIHS
Mathematical modelling	August 2018	ISPM, Bern
*Publication of mathematical mo*del	September 2018	

Part – IV

Ethical Consideration

22. Regarding the human participants:

Are human participants required in this research? If yes, provide justification.

[X] Yes (*provide justification*) No [ ]

Screening echocardiography will be performed among school going children 5 to 15 years of age since the occurrence of rheumatic fever ist highest in this age group and early detection is expected to have to greatest benefit on future prognosis. Screening echocardiography is a non-invasive examination without radiation exposure that is not associated with any harmful complications.

How many participants are required for the research? Explain.

We calculated a sample size of 9500 schoolchildren between the ages of 5 to 15 years with a type I error of 0.05 and a power of 90% for an expected diagnosis rate of 2 per 1000.

What is the frequency of the participant's involvement in the research? Explain.

All children with documented history of ARF and/or echocardiographic evidence of RHD will be re-examined in regular time intervals, at six months, at one year, and yearly thereafter up to at least 5 years, in the context of a prospective cohort study. Both children and their primary caregivers will be educated in order to ensure compliance with secondary prevention and regular follow-up. A standardized questionnaire will address clinical symptoms, compliance to treatment, and assess pre-specified clinical endpoints. Echocardiographic follow-up will be performed yearly up to five years at B.P. Koirala Institute of Health Sciences (BPKIHS).

Clearly indicate the participant's responsibilities in the research. What is expected of the research participants during the research?

We anticipate that compliance with secondary prevention will be one of the major challenges, which in turn requires continued education. We plan to ensure compliance by means of face-to-face education by the research coordinator at each visit to both the child and the caregiver, making them aware of the risk of recurrence of rheumatic fever and the potential consequences of progression of rheumatic heart disease. Visit reminders by mail and if necessary by personal visits will also be used to ensure adequate compliance.

Are vulnerable members of the population required for this research? If yes, provide justification.

Screenng examinations will be performed in schoolchildren since the prevalence of silent rheumatic heart disease and therefore the burden of still preventable morbidity and mortality is greatest among children 5-15 years of age.

Are there any risks involved for the participants? If yes, identify clearly what are the expected risks for the human participants in the research and provide a justification for these risks.

Primary caregivers will be educated to detect symptoms and signs of potential allergic reactions of antibiotic treatment such as a skin rash, hives, swollen lips/tongue and wheezing , and will be provided with an emergency medical contact number. In case of drug-related adverse side effects such as diarrhea, nausea, pain/swelling/infection at the site of injection, we will recommend to switch the antibiotic administration from intramuscular to oral or vice versa as a first step. If children/caregivers refuse regular antibiotic intake despite counseling, they will have the option to retract their decision at any given point in time. With their consent, these children will remain in the registry in order to provide care and treatment in case of potential late-complications as a consequence of disease progression. In the event of children being

diagnosed at an advanced stage with significant valvular heart disease leading to congestive heart failure, we will assure optimal interdisciplinary medical treatment.

Are there any benefits involved for the participants? If yes, identify clearly what are the expected benefits for the participants.

Early implementation of secondary antibiotic prevention my prevent progression of rheumatic heart disease.

23. Informed Consent Form / Ethical Issues:

Statements required in the Informed Consent Form include:

A statement that the human participants can withdraw from the study at any time without giving reason and without fear. State clearly how the participants can opt out the study.

A statement guaranteeing the confidentiality of the research participants.

If required, a statement on any compensation that might be given to the research participant and or their community.

A statement indicating that the participants has understood all the information in the consent form and is willing to volunteer / participate in the research.

Signature space for the research participants, a witness, and the date.

(Informed Consent form should be submitted in English and in the language appropriate to the research participants)

Obtaining the Consent
How informed consent is obtained from the research participants?

[ ] Verbal [x] Written

Please indicate who is responsible for obtaining informed consent from the participants in this research study?

The local study coordinator will collect the consent from the parents of the children.

Is there anything being withheld from the research participants at the time the informed consent is being sought?

No, there is nothing withheld from the research participants.

If yes, explain

....

Is the research sensitive to the Nepali culture and the social values?

Yes [✓] No [ ] Explain.

....

Is health insurance *(if applicable)* being made available to the research participants? If yes, please provide the necessary insurance data.

There is no particular health insurance available to the research participants, however, secondary antibiotic prevention as well as follow-up examinations will be for free for the study participants.

(Include in consent form)

24. Regarding Clinical Trial:

The study is not a clinical trial, but a cross-sectional survey with a subsequent registry of patients with rheumatic heart disease.

In case of a clinical trial address the following:
The trial treatment

....

A detailed explanation of the trial procedures including all invasive procedures.

....

The potential or direct benefits (if any) for the research participants.

....

Alternative procedure(s) or treatment(s) that may be available.

....

The risks, discomforts, and inconveniences associated with the study

....

Provisions for management of any adverse reactions

....

The provisions of insurance coverage for any permanent disability or death caused directly by the investigational treatment or procedure.

....

The provision of including the name and address, including telephone numbers of person to be contacted in case of adverse events or for any information related to the trial.

....

Is there going to be a transfer of any biological materials from the country? Explain.

....

Is there a Data Safety Monitoring Board?
If Yes, Mention

....

Is this trail internationally registered?
Yes, the trial was registered with 'www.ClinicalTrials.gov' (NCT01550068)......................................

Part – V

ACCEPTANCE OF GENERAL CONDITIONS AND DECLARATION BY THE PRINCIPAL INVESTIGATOR

I hereby certify that the above mentioned statements are true, I have read and understood the regulation of the Nepal Health Research Council (NHRC) on the approval of research proposal and will act in conformity with the said regulation in all respects.

If the research is terminated, for any reason, I will notify NHRC of this decision and provide the reasons for such actions. I will provide NHRC with a written notice upon the completion of the research as well as a final summary/full report of the research study. If I publish the results in a journal, I shall acknowledge the NHRC and shall provide the Council with three copies of any such articles.

Dr. med. Thomas Pilgrim
Oberarzt Invasive Kardiologie
Universitätsspital Bern
3010 Bern
Tel. 031/632 21 11/PSA 7988

....

Signature of Applicant

Oct 27 2012

Date: ....

INFORMED CONSENT:

- Describe the manner in which informed consent will be obtained.
- Indicate what kind of consent (e.g. parental, child, adult, etc) will be used.
- If the subjects are children/adolescents ages 7-18 years, an Assent Form must be included with the IRB application. The signed Assent Form along with the Parental/Guardian Consent Form must be retained on file for at least three years after completion of the research project.
- If prisoners / pregnant women, or fetuses are to be included in the research sample, it is likely that a full IRB review will be required and additional human subjects' protections will be expected.
- If the subjects do not read or comprehend English, you must provide a consent form in their language as well as in English for IRB review and approval.
- If you are requesting a waiver of written consent (i.e. a signature on an informed consent form) from the subjects, you MUST justify this request by providing an explanation of why obtaining written consent would add additional risk to the subjects and your alternative provisions for informing them about the study.
- If consent documents from another site will be used, you will have to indicate this and provide a copy of the authorized consent document and IRB approval with your application.
- You will have to provide any other relevant information if necessary. Please be aware that the PI is legally required to retain all signed Informed Consent forms for at least three years after the project terminates
- The Informed Consent form must be written at a level that the subjects will understand. Please use simple language, and avoid clinical jargon.
- Attach a copy of the written informed consent form (assent or parental consent where applicable). Consent documents MUST be in format requested. See examples on line.
- If the study uses database or archival data the use of informed consent is not applicable.

CONFIDENTIALITY OF DATA: ***Confidentiality of data MUST be address for all studies.***

- Indicate the extent to which confidentiality of records identifying subjects will be maintained.
- Describe the storage and disposal of information where applicable.

Check List

For all applicants

1. Covering letter addressed to the Member secretary indicating the submission of the approval of proposal.
2. Proposal will only be accepted if submitted in NHRC format.
3. Both printed and electronic version of the proposal should be submitted.
4. Curriculum Vitae of the Principal Investigator & Co-Principal Investigator of the study team should be submitted.
5. If the Principal Investigator is a non Nepali citizen, at least one Co-investigator should be a Nepali citizen.
6. Submission of the application processing fee to NHRC.(According to NHRC rules and regulations)
7. Source of funding for the proposed project.
8. The proposal should have institutional ethical clearance from his/her own country if submitted from academic and related institution.
9. If the research study is to be conducted in any hospitals/organization or institution/community, a letter of approval from the related hospital/organization or institution/district authority should be provided.
10. Consent form should be in Nepali & local language (if necessary).
11. Data collection tools should be in Nepali & local language (if necessary) including interview guideline, observation checklist, questionnaires etc.
12. Style of referencing should be in Harvard style.
13. List of abbreviations / acronyms should be provided.

For students' applicants

1. Approval letter from concern Institute/University.
2. Recommendation letter from Academic Supervisor.

Processing Fee

Researcher has to pay the processing fee as per the rules and regulations of NHRC.

Nepal Health Research Council
Estd. 1991

NHRC

14 August 2013

Ref. No. 206

Executive Committee

Executive Chairman
Prof. Dr. Chop Lal Bhusal

Vice - Chairman
Dr. Rishi Ram Koirala

Member-Secretary
Dr. Shanker Pratap Singh

Members
Prof. Dr. Meeta Singh
Prof. Dr. Suman Rijal
Dr. Narendra Kumar Singh
Dr. Samjhana Dhakal
Dr. Devi Gurung

Representative
Ministry of Finance
National Planning Commission
Ministry of Health & Population
Chief, Research Committee, IOM
Chairman, Nepal Medical Council

Dr. Thomas Michael Pilgrim
Principal Investigator
Swiss Cardiovascular Center,
Bern University Hospital, Switzerland

Subject: Regarding comments and suggestions of the research proposal entitled **population- based study of rheumatic heart disease prevalence and cardiovascular outcomes among schoolchildren in Nepal**

Dear Dr. Pilgrim,

Please find the attached copy of the Comments and Suggestions provided by the experts of the above- mentioned proposal. We would like to request you to go through these suggestions and submit it accordingly.

Looking forward for your prompt response.

Thanking you.

Dr. Shanker Pratap Singh
Member- Secretary

Comments/Suggestions

1. Aim of the research as the authors have described is not to evaluate or develop a protocol but it is to conduct a study on RHD prevalence and evaluate the cardiovascular outcome of secondary prevention in eastern Nepal. It would be appropriate to entitle the research as population- based study of RHD prevalence and cardiovascular outcomes of secondary prevention among schoolchildren in a selected district of eastern Nepal. The list of variables does not seem enough by seeing the objectives. Please list them and use this to depict their hypothesized and use this to depict their hypothesized relationship in conceptual framework.
2. You are requested to clarify the duration of follow up (3 or 5 years) in the longitudinal cohort study (in the text 5 years is mentioned but the work plan shows 3 years)
3. Data collection tools are good. It is suggested to include arthritis also along with other major manifestations in the physical examine section of the case report form-observation survey.
4. However, by seeing the large sample size you are planning to cover, it looks like you could make the study representative of eastern Nepal by slightly changing the sample design.
5. Will there be any way to have a comparison group for the longitudinal study. It would enhance the scientific strength of the study.
6. Please elaborate the data collection technique for the follow up component.
7. As this is a study being carried out by two academia, as a capacity-building component, it could be advisable to enroll some body for higher degrees (for e.g. PhD).

INSELSPITAL
UNIVERSITÄTSSPITAL BERN
HOPITAL UNIVERSITAIRE DE BERNE

August 17, 2013

Swiss Cardiovascular Center Bern
University Hospital
Bern, Switzerland

Chairman: Prof. Bernhard Meier

Chief-of-Cardiology: Prof. Stephan Windecker

phone: (+41 31) 632 44 97
fax: (+41 31) 632 11 31
e-mail stephan.windecker@insel.ch

Population-based Study of Rheumatic Heart Disease Prevalence and Cardiovascular Outcomes of Secondary Prevention among Schoolchildren in a Selected District of Eastern Nepal

Dear Dr. Shanker Pratap Singh,

thank you for the careful review of the above mentioned research protocol and your valuable suggestions.

The research protocol has been revised according to your suggestions. We have addressed each of your comments in a separate rebuttal letter. We think that the revised version of the research protocol has been further improved and hope to have satisfactorily addressed all of the raised issues.

We thank you to reconsider the revised protocol for approval by the Nepal Health Research Council.

We thank you to consider our proposal for approval.

Sincerely,

Thomas Pilgrim, MD

Rebuttal Letter

Population-based Study of Rheumatic Heart Disease Prevalence and Cardiovascular Outcomes of Secondary Prevention among Schoolchildren in a Selected District of Eastern Nepal

Comment 1a: Aim of the research as the authors have described is not to evaluate or develop a protocol but it is to conduct a study on RHD prevalence and evaluate the cardiovascular outcome of secondary prevention in eastern Nepal. It would be appropriate to entitle the research as population-based study of RHD prevalence and cardiovascular outcomes of secondary prevention among schoolchildren in a selected district of eastern Nepal.

Reply 1a: We thank you for this comment. According to your suggestion we entitle the research project "population-based study of RHD prevalence and cardiovascular outcomes of secondary prevention among schoolchildren in a selected district of eastern Nepal".

Comment 1b: The list of variables does not seem enough by seeing the objectives. Please list them and use this to depict their hypothesized relationship in conceptual framework.

Reply 1b: We thank you for this comment and extended the list of variables as follows:

Baseline variables –Observational Survey:

- Date of Birth
- Initials
- Gender
- Date of Screening
- School
- Grade, Class
- Number of family members, Number of children/adults in the household
- Number of rooms in the house
- Number of cars/motorbikes owned by parents
- Number of classmates
- Television at home
- Numbers of books at home (0, <10, 10-100, >100)
- Personal cellular phone
- Access to internet connection at home
- Age of primary caregiver
- Education of primary caregiver (profession of honours, graduate/postgraduate, post highschool diploma, high school vertificate, middle school certificate, primary school/literate, illiterate)
- Occupation of primary caregiver (profession, semi-profession, clerical/show-owner/farmer, skilled worker, semi-skilled worker, unskilled worker, unemployed,
- Estimated family income per month
- Type of house (Kachcha, Tin, Wooden, Pakka)
- Previous history of rheumatic fever
- Joint pain, Arthralgia
- Height, Weight, Waist circumference
- Erythema marginatum
- Subcutaneous nodules
- Arthritis
- Chorea
- Heart murmur

Primary Endpoints Observational Survey:
WHF Criteria for Echocardiographc diagnosis of RHD
Definite RHD

- A) Pathological MR and at least two morphological features of RHD of the MV
- B) MS mean gradient greater or equal 4 mmHg
- C) Pathological AR and a t least two morphological features of RHD of the AV
- D) Borderline disease of both the AV and the MV

Borderline RHD

- A) At least two morphological features of RHD of the MV without pathological MR or MS

- B) Pathological MR
- C) Pathological AR

Modified WHO Criteria
- Definite RHD: Cardiac murmur and significant MR and/or significant AR and a thickened MV and/or elbow deformity of the anterior mitral leaflet
- Probable RHD: Cardiac murmur and either significant MR and(or AR or thickened MV and/or elbow deformity of the anterior mitral leaflet
- Possible RHD: No cardiac murmur and thickened mitral valve and/or elbow deformity of mitral valve and/or significant MR and/or AR

<u>Follow-up variables –Cohort Study:</u>
- Date of Birth
- Initials
- Gender
- Date of Follow-up
- Antibiotic regiment for secondary prevention (no secondary prevention/peniciliiin G benzathine i.m., Penicillin V p.o., Sulfadiazine p.o., Azithrombyzin, other)
- Height
- Weight
- Waist circumference
- Heart murmur

<u>Primary Endpoints – Cohort Study:</u>
Primary Clinical Endpoints:
- Death
- Stroke
- TIA
- Infectious endocarditis
- Hospitalisation for congestive heart failure
- Valvuar heart surgery
- Mitral balloon valvuloplasty
- Recurrence of rheumatic fever
-

Primary Echocardiographic Endpoints:
WHF Criteria for Echocardiographc diagnosis of RHD
Definite RHD
- A) Pathological MR and at least two morphological features of RHD of the MV
- B) MS mean gradient greater or equal 4 mmHg
- C) Pathological AR and a t least two morphological features of RHD of the AV
- D) Borderline disease of both the AV and the MV

Borderline RHD
- A) At least two morphological features of RHD of the MV without pathological MR or MS
- B) Pathological MR
- C) Pathological AR

Modified WHO Criteria
- Definite RHD: Cardiac murmur and significant MR and/or significant AR and a thickened MV and/or elbow deformity of the anterior mitral leaflet
- Probable RHD: Cardiac murmur and either significant MR and(or AR or thickened MV and/or elbow deformity of the anterior mitral leaflet
- Possible RHD: No cardiac murmur and thickened mitral valve and/or elbow deformity of mitral valve and/or significant MR and/or AR

The documentation of a wide array of baseline variables will give us a more accurate understanding of the population at risk and help us to identify determinants of compliance to secondary prevention and eventually disease progression. We hypothesize, that lower education of the parents, lower economic status, poorer housing and overcrowding are associated with a higher prevalence of RHD.
For the cohort study, the precise documentation of clinical findings and echocardiographic lesions according to the criteria provided by the World Heart Federation at baseline and during follow-up will allow us to investigate the importance of age and stage of disease at presentation, risk factors of disease

progression, and efficacy of secondary prevention for subclinical RHD. We hypothesize, that both older age at diagnosis and more advanced stage of disease at presentation are correlated with more rapid disease progression and adverse clinical outcome.

Comment 2: You are requested to clarify the duration of follow-up (3 or 5 years) in the longitudinal cohort study. (in the text: 5 years is mentioned but the work plan shows 3 years).

Reply 2: We thank you for making us aware of this inconsistency. The duration of the longitudinal cohort study will be 5 years. More events collected in 5 instead of 3 years will increase the scientific strength to predict progression of disease.

Comment 3: Data collection tools are good. It is suggested to include arthritis also along with other major manifestations in the physical examine section of the case report form – observation survey.

Reply 3: We thank you for this comment and agree that arthritis should be included along the other major manifestations of rheumatic fever.

Comment 4: However, by seeing the large sample size you are planning to cover, it looks like you could make the study representative of eastern Nepal by slightly changing the sample design.

Reply 4: We agree that the study population should be representative for eastern Nepal. According to the most recent data provided by the United Nations from the year 2011, 83% of the population of Nepal lives in rural areas (Source: http://data.un.org/CountryProfile).
Among a total of 595 schools in the Sunsari district, 503 (85%) are located in rural areas whereas 92 (15%) are located in urban areas. In addition, 383 (64%) out of the 595 schools are governmental schools, and 122 (36%) are private schools. In order to reflect this distribution we decided to select schools in a rural to urban ratio of 3:1, and a governmental to private ratio of 2:1. Based on these proportions, we come to a lowest common denominator of 12 schools. A modification of this sample design would increase the lowest common denominator and make an estimation of the total number of children to be included into the observational survey almost impossible. An additional source of imprecision is the fact, that the number of students per school is not implemented in the sample size calculation. Therefore, we think that the sample design we applied is a realistic compromise between accuracy and practicability and provides a reasonable approximation to the population distribution in the Sunsari district. However, if you should disagree with our suggestion to maintain the current sample design or propose an alternative sample model, we will be happy to reconsider the current design.

Comment 5: Will there be any way to have a comparison group for the longitudinal study. It would enhance the scientific strength of the study.

Reply 5: We agree that a comparison group for the longitudinal cohort study would enhance the scientific strength of the study. We therefore extended the current design to a cluster randomized trial in which one school reflects one cluster.
All schools in the Sunsari district are categorized in one out of four groups: 1. rural governmental 2. rural non-governmental 3. urban non-governmental 4. urban governmental. All schools within one group are sorted according to a randomly assigned individual number (e.g. 1-503) using research randomizer (http://www.randomizer.org/). Based on the randomly assigned increasing numbers there will be a list of schools in each group. Starting from the top of the list, 12 schools from the 4 groups will be selected in a 6:3:2:1 proportion as outlined above. All children from one of those 12 selected schools (A1-A12) will then be screened for RHD at timepoint A. The next 12 schools on the randomly created lists will then be selected as the control group (A13-A24). After three years (timepoint B), all 24 schools will be revisited and all children will be screened by echocardiography. Using this approach, at timepoint B burden of morbidity among children aged 8-15 in schools A1-A12 can be compared to burden of RHD morbidity among children aged 8-15 in schools A13-A24 at timepoint B. Children younger than 8 years of age will not qualify for inclusion into this comparison, since they were not potential candidates for screening at timepoint A.

Comment 6: Please elaborate the data collection technique for the follow-up component.

Reply 6: We agree that the data collection technique for the follow-up component should be outlined in more detail and therefore added the following paragraph in the revised version of the proposal.
All children with valvular lesions consistent with RHD will be included into a prospective registry. The parents or caregivers will be contacted and invited to BPKIHS outpatient clinic for detailed information

on RHD and education on the importance of secondary prevention. Secondary antibiotic prevention will be provided free of charge every 3-4 weeks at BPKIHS. After informed consent, clinical and echocardiographic follow-up will be organized on a regular basis after 6 months, after 1 year and yearly thereafter until 5 years. Parents of the children will be reminded of the follow-up visits by telephone calls. The following variables will be collected during follow-up:

Follow-up variables –Cohort Study:

- Date of Birth
- Initials
- Gender
- Date of Follow-up
- Antibiotic regiment for secondary prevention (no secondary prevention/peniciliiin G benzathine i.m., Penicillin V p.o., Sulfadiazine p.o., Azithrombyzin, other)
- Height
- Weight
- Waist circumference
- Heart murmur

Primary Endpoints – Cohort Study:
Primary clinical endpoints:

- Death
- Stroke
- TIA
- Infectious endocarditis
- Hospitalisation for congestive heart failure
- Valvuar heart surgery
- Mitral balloon valvuloplasty
- Recurrence of rheumatic fever
-

Primary Echocardiographic Endpoints:
WHF Criteria for Echocardiographc diagnosis of RHD
Definite RHD

- A) Pathological MR and at least two morphological features of RHD of the MV
- B) MS mean gradient greater or equal 4 mmHg
- C) Pathological AR and a t least two morphological features of RHD of the AV
- D) Borderline disease of both the AV and the MV

Borderline RHD

- A) At least two morphological features of RHD of the MV without pathological MR or MS
- B) Pathological MR
- C) Pathological AR

Modified WHO Criteria

- Definite RHD: Cardiac murmur and significant MR and/or significant AR and a thickened MV and/or elbow deformity of the anterior mitral leaflet
- Probable RHD: Cardiac murmur and either significant MR and(or AR or thickened MV and/or elbow deformity of the anterior mitral leaflet
- Possible RHD: No cardiac murmur and thickened mitral valve and/or elbow deformity of mitral valve and/or significant MR and/or AR

Variables collected during follow-up will be entered into a web-based data entry system (REDCap) hosted at the Clinical Trials Unit at Bern University Hospital.

Comment 7: As this is a study being carried out by two academia, as a capacity-building component, it could be advisable to enroll somebody for higher degrees (for e.g. PhD).

Reply 7: We acknowledge that capacity-strengthening will be of key importance throughout the course of the study and are making considerable efforts to guarantee sustainability of the project. Capacity-strengthening will occur on different levels. First, selected fellows from BPKIHS involved in the screening process will be trained in echocardiography using portable ultrasound devices. Second, the Department of Cardiology at the University of Bern will provide clinical proctoring for invasive treatment strategies required for children identified with cardiac disease during screening (mitral balloon valvuloplasty, ASD

closure). Dr Nikesh Shrestha has already completed an interventional cardiology fellowship program at Bern University between January and September 2010 which formed the basis for a continued exchange between the two institutions. This transfer of knowledge will be the basis for a sustainable program. Third, study personnel will be educated on good clinical practice (GCP) and instructed on database management and statistical analysis.

We will be open to enroll an interested fellow for higher degrees and will be happy to involve local students and residents to perform sub-analyses of the database and will assist in providing support where needed.

Research Proposal Approval Format

**Research Title:**
**Population-based Study of Rheumatic Heart Disease Prevalence and Cardiovascular Outcomes among Schoolchildren in Nepal**

Nepal Health Research Council (NHRC)

P.O. Box: 7626, Ramshah Path, Kathmandu, Nepal

Tel: +977-1-4254220, 4227460, Fax: +977-1-4262469

E-mail: nhrc@nhrc.org.np, Website: http://www.nhrc.org.np

For Official Use Only

(Please see the check list before Registration of the application form)

Registration No.:
Registration Date:
Approved Date:
Name of PI:
Total Budget of the Project:
NHRC Processing Fee:
Research Site:
Tentative Date of Initiating the Project:
Duration of the Research Project:
Name of Internal Reviewer:
Name of External Reviewer:
Signature & Seal of NHRC:

Part – I

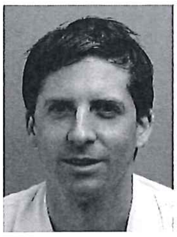

Administrative Information

1. Research Title: **Population-based Study of Rheumatic Heart Disease Prevalence and Cardiovascular Outcomes of Secondary Prevention among Schoolchildren in a Selected District of Eastern Nepal**

2. Name and Title of Principal Investigator responsible for the proposed research:

Pilgrim	Michael	Thomas
Last (Surname)	Middle (if any)	First name

Nationality: Swiss

Citizenship Number with district name from where it was obtained (only for Nepali)

Passport Number (only for non Nepali citizen): F0265380

Signature: [illegible] Date: 17.08.2013

Postal Address: Freiburgstrasse 10, CH-3010 Bern, Switzerland

Telephone No.: +41 31 632 21 11

Mobile No.: +41 76 548 44 11

Fax No.: +41 31 632 47 70

e-mail: thomas.pilgrim@insel.ch

Alternate e-mail: pilgrimthomas@hotmail.com

3. Full name of the Institution associated with the Principal Investigator (if applicable) : Swiss Cardiovascular Center, Bern University Hospital, Switzerland

Designation: Attending Physician

Postal Address (if different from the address given above):

Telephone No.:

Fax No.:

e-mail:

Website:

4. Declaration of the head of the Institution (if applicable)

If the proposed research is approved, we will allow him/her to conduct the research in this institution.

Signature: Date:

Gupta	Kumar	Arvind
Last (Surname)	Middle (if any)	First name

Designation: Hospital Director

Name of the Institution: B. P. Koirala Institute of Health Sciences

Contact/Postal Address: Dharan

Telephone No.: 025-525555

Fax No.:

Institutional e-mail:

Website: www.bpkihs.edu.np

5. Name and Title of Co-investigators responsible for the proposed research (Use the similar format if more than one):

Passport size photograph

Shrestha	Raj	Nikesh
Last (Surname)	Middle (if any)	First name

Nationality: Nepali

Citizenship Number with district name from where it was obtained (only for Nepali)

Passport Number (only for non Nepali citizen):

Affiliated Institution (if applicable): BPKIHS

Designation: Associate Professor

Signature: Date:

Postal Address (if different from the address given above):

Telephone No.:
Fax No.:

e-mail:

6. Name and Title of Co-investigators responsible for the proposed research (Use the similar format if more than one):

Passport size photograph

Agrawaal	Kumar	Krishna
Last (Surname)	Middle (if any)	First name

Nationality: Nepali

Citizenship Number with district name from where it was obtained (only for Nepali) 55071 NawalParasi

Passport Number (only for non Nepali citizen):

Affiliated Institution (if applicable): BPKIHS

Designation: Senior Resident

Signature: Date:

Postal Address (if different from the address given above):

Telephone No.:
Fax No.:

e-mail: agrawalkris@gmail.com

7. Name and Title of Co-investigators responsible for the proposed research (Use the similar format if more than one):

Karki		Prahlad
Last (Surname)	Middle (if any)	First name

Passport size photograph

Nationality: Nepali

Citizenship Number with district name from where it was obtained (only for Nepali):

Passport Number (only for non Nepali citizen):

Affiliated Institution (if applicable):

Designation: Professor and Head

Signature: Date:

Postal Address (if different from the address given above):

Telephone No.:

Fax No.:

e-mail:

(Use additional sheet if necessary)

8. List the name(s) and institutional affiliation to the researcher(s) (other than co-investigator) to assist your project in Nepal and abroad (if any)

	Name	*Institution and Address*
(a)	B.P. Koirala Institute of Helath Sciences	
(b)	Swiss Cardiovascular Center, Bern	

(Use additional sheet if necessary)

9. List the name(s) of Nepali researcher(s) (other than co-investigator) or Nepalese Institution/hospital/NGO(s) etc. from whom you may seek co-operation (if any)

(a)

(b) [ ]

(Use additional sheet if necessary)

8. List major equipment(s) in relation to your research project you plan to bring/import to Nepal (If applicable)

(a) [ ]

(b) [ ]

(Use additional sheet if necessary)

8.1 List details of all specimen(s) (if any) that you may transport from Nepal in relation to your research.

(a) [ ]

(b) [ ]

(c) [ ]

(d) [ ]

8.2 Country of Destination: [ ]

Name of Institution: [ ]

8.3 Mode of Transportation of Specimen [ ]

8.4 How will you ensure duplicate specimens remain in the country?

[ ]

(If necessary use additional sheet)

[ ]

9. Is this research part of your Thesis?

Yes [ ] No [x]

If yes,

For what degree and in which subject? [ ]

From which university? [ ]

From which country?

Part – II

Financial Information

10.Research Title:

Population-based Study of Rheumatic Heart Disease Prevalence and Cardiovascular Outcomes of Secondary Prevention among Schoolchildren in a Selected District of Eastern Nepal

11.Name of the funding organization:

Fondation de la Tour, Meyrin-Geneva, Switzerland

Contact information of funding organization or agency:

Postal Address: Av-Maillard 1, CH-1217 Meyrin-Geneva, Switzerland

Telephone No.:

Fax No.:

e-mail: purban@latour.ch

Contact person at the funding organization or agency:

Urban		Philip
Last (Surname)	Middle (if any)	First name

Designation: President of the Foundation

Total amount of funds (in NRs / US $) allocated for the proposed research project: 9885 US $

Itemized budget (in detail) and justify the resources required for the proposed research work (*use additional sheet*)

Transportation Charges: 1070 US$

OPD Ticket charge: 615 US$

Logistics: 800 US$

Salary: 6200 US$

Miscellaneous: 1200 US$

Part – III

Research Proposal Description

12.Research Title:

Population-based Study of Rheumatic Heart Disease Prevalence and Cardiovascular Outcomes of Secondary Prevention among Schoolchildren in a Selected District of Eastern Nepal

13.Proposal Summary (maximum 500 words):

Introduction: Rheumatic heart disease (RHD) remains a major contributor to morbidity and mortality in developing countries. The reported prevalence rates of RHD are highly variable and mainly attributable to differences in the sensitivity of either clinical screening to detect advanced heart disease, or echocardiographic evaluation where disease is diagnosed earlier across a continuous spectrum. The clinical significance of diagnosis of subclinical RHD by echocardiographic screening and early implementation of secondary prevention has not been clearly established.

Methods and Analysis: We designed a cross-sectional survey to determine the prevalence of RHD in children from randomly selected private and public schools between the age of 5 and 15 years in urban and rural areas of Eastern Nepal using both cardiac auscultation and echocardiographic evaluation. Children with RHD will be treated with secondary prevention and enrolled in a prospective cohort study. A second badge of randomly selected schools will undergo screening later in the course of the study and act as a control group. We will compare the prevalence rates by cardiac auscultation and echocardiography, determine risk factors associated with diagnosis and progression of RHD, investigate social and economic barriers for receiving adequate cardiac care, and assess clinical outcomes with regular medical surveillance

as a function of stage of disease at the time of diagnosis. Prospective clinical studies investigating the impact of secondary prevention for subclinical RHD on long-term clinical outcome will be of central relevance for future health resource utilization in developing countries.

Ethics and Dissemination: The study was considered ethically uncritical and was given an exempt status by the ethics committee at University of Bern, Switzerland. The study has been registered with 'www.ClinicalTrials.gov' (NCT01550068). The study findings will be reported in peer-reviewed publications.

14. Introduction:

14.1 Background of Study (maximum 500 words):

Rheumatic fever complicated by rheumatic heart disease (RHD) remains a major contributor to morbidity and premature death among the socio-economically underprivileged working age population of developing countries. RHD results from an autoimmune response due to molecular mimicry between the M-protein on the group A β-hemolytic streptococci cell membrane and cardiac myosin eventually leading to valvular damage. High prevalence rates of acute rheumatic fever (ARF) and RHD have been reported from different geographic regions around the world such as Southeast Asia, the Western Pacific and Africa that share demographic characteristics determined by poverty and limited access to health care resources. The burden of RHD is likely to escalate in these countries due to increasing urbanization and overcrowding.

Prevalence rates of RHD from screening studies in Southeast Asian countries range from 0.7 to 22 per 1000 children using traditional cardiac auscultation, and from 20 to 51 per 1000 children using echocardiography. A considerable variation in prevalence rates reflects the substantially higher sensitivity of echocardiographic screening as compared to cardiac auscultation due to diagnosis across a continuous spectrum of disease as opposed to presence or absence of a heart murmur using cardiac auscultation. Echocardiographic screening for RHD has been recommended by the World Health Organization (WHO) in high prevalence regions, and the recently released criteria for echocardiographic diagnosis of RHD by the World Heart Federation (WHF) warrant consistent reporting and facilitate the evaluation of progression of minor echocardiographic lesions over time. The diagnosis of RHD at an earlier, clinically silent stage by the detection of morphological and functional valvular lesions without a corresponding heart murmur challenges our current concept of prevention and treatment.

Medical management of ARF and RHD largely depends upon preventive measures comprising reduction of overcrowding, prompt antibiotic treatment of streptococcal pharyngitis, and secondary prevention achieved by regular oral or intramuscular administration of penicillin continued until early adulthood among children with a documented history of ARF or evidence of RHD. Even though preventive measures with penicillin are inexpensive and efficient, this strategy is difficult to effectuate in developing countries with limited access to health care resources. Enrollment of patients with ARF and RHD in a

registry with close follow-up has been demonstrated to reduce the cardiovascular sequelae associated with disease progression.

14.2 Statement of the Problem and Rationale / Justification (maximum 500 words)

Effective measures to reduce the global burden of RHD represent an ongoing challenge involving reduction in overcrowding, improving hygiene, increasing public awareness and facilitating access to health care. In the absence of fundamental socio-economic changes improving primordial prevention, systematic screening for RHD based on public and private education represents the most comprehensive approach and aims at a reduction of the late complications of RHD by early implementation of secondary prevention. Current research has been predominantly focused on assessing prevalence rates using passive survey systems without subsequent enrolment in registries or offering longitudinal follow-up. In order to assess the determinants of disease and its progression along with short- and long-term clinical outcomes, we plan to include all cases of RHD in a cohort study to be treated according to their disease stage at diagnosis and followed up for at least 5 years.

14.3 Conceptual framework

The screening part of the study will be conducted for a period of 12 months. Governmental and non-governmental schools from rural and urban areas in the Sunsari district will be selected for screening in agreement with the district education office. All schoolchildren 5 to 15 years of age will be eligible for screening if parents do not withdraw consent for screening. A questionnaire designed to assess demographic characteristics and socio-economic parameters will be completed for all children. Following a short physical examination, a focused screening echocardiography will be performed to detect rheumatic valvular lesions according to the definitions provided by the World Heart Federation. Parents of children with evidence of RHD will be contacted by the physicians and oriented about the findings. The children and parents will subsequently be invited for a thorough examination at B.P. Koirala Institute of Health Sciences. If rheumatic heart disease is confirmed in a repeat

echocardiography, secondary antibiotic prevention with regular intramuscular penicillin injections will be recommended. Collaboration with family physicians and pediatricians will be central to improve long-term adherence to secondary antibiotic prevention for silent rheumatic heart disease. After informed consent for inclusion into a prospective registry, follow-up data of children with borderline or definite RHD will be collected during a dedicated follow-up visit after six months, at one year and yearly thereafter up to five years.

14.4 Research Objectives / purpose / aim of the study:

General

The objective of this study is to investigate the prevalence rate of definite and borderline RHD among children in Eastern Nepal, and to assess long-term clinical outcome of children undergoing secondary antibiotic prevention for RHD.

Specific

More specifically, we aim to (a) compare the prevalence rates by cardiac auscultation and echocardiography, (b) determine risk factors associated with diagnosis and progression of RHD, (c) investigate social and economic barriers for receiving adequate cardiac care, and (d) assess clinical outcomes as a function of stage of disease at the time of diagnosis with regular medical surveillance.

15. Research Design and Methodology

Research Method

Qualitative ☐ Quantitative ☐ Combined ☑

Study Variables:

Baseline variables –Observational Survey:

- Date of Birth
- Initials
- Gender
- Date of Screening
- School
- Grade, Class
- Number of family members, Number of children/adults in the household
- Number of rooms in the house
- Number of cars/motorbikes owned by parents
- Number of classmates
- Television at home
- Numbers of books at home (0, <10, 10-100, >100)
- Personal cellular phone
- Access to internet connection at home
- Age of primary caregiver
- Education of primary caregiver (profession of honours, graduate/postgraduate, post highschool diploma, high school vertificate, middle school certificate, primary school/literate, illiterate)
- Occupation of primary caregiver (profession, semi-profession, clerical/show-owner/farmer, skilled worker, semi-skilled worker, unskilled worker, unemployed,
- Estimated family income per month
- Type of house (Kachcha, Tin, Wooden, Pakka)
- Previous history of rheumatic fever
- Joint pain, Arthralgia
- Height, Weight, Waist circumference
- Erythema marginatum
- Subcutaneous nodules
- Arthritis
- Chorea
- Heart murmur

Primary Endpoints Observational Survey:
WHF Criteria for Echocardiographc diagnosis of RHD
Definite RHD

- A) Pathological MR and at least two morphological features of RHD of the MV
- B) MS mean gradient greater or equal 4 mmHg
- C) Pathological AR and a t least two morphological features of RHD of the AV
- D) Borderline disease of both the AV and the MV

Borderline RHD

- A) At least two morphological features of RHD of the MV without pathological MR or MS
- B) Pathological MR
- C) Pathological AR

Modified WHO Criteria

- Definite RHD: Cardiac murmur and significant MR and/or significant AR and a thickened MV and/or elbow deformity of the anterior mitral leaflet
- Probable RHD: Cardiac murmur and either significant MR and(or AR or thickened MV and/or elbow deformity of the anterior mitral leaflet
- Possible RHD: No cardiac murmur and thickened mitral valve and/or elbow deformity of mitral valve and/or significant MR and/or AR

Follow-up variables –Cohort Study:
Date of Birth
Initials
Gender
Date of Follow-up
Antibiotic regiment for secondary prevention (no secondary prevention/peniciliiin G benzathine i.m., Penicillin V p.o., Sulfadiazine p.o., Azithrombyzin, other)

- Height
- Weight
- Waist circumference
- Heart murmur

Primary Endpoints – Cohort Study:
Primary Clinical Endpoints:

- Death
- Stroke
- TIA
- Infectious endocarditis
- Hospitalisation for congestive heart failure
- Valvuar heart surgery
- Mitral balloon valvuloplasty
- Recurrence of rheumatic fever

Primary Echocardiographic Endpoints:
WHF Criteria for Echocardiographc diagnosis of RHD
Definite RHD

- A) Pathological MR and at least two morphological features of RHD of the MV
- B) MS mean gradient greater or equal 4 mmHg
- C) Pathological AR and a t least two morphological features of RHD of the AV
- D) Borderline disease of both the AV and the MV

Borderline RHD

- A) At least two morphological features of RHD of the MV without pathological MR or MS
- B) Pathological MR
- C) Pathological AR

Modified WHO Criteria

- Definite RHD: Cardiac murmur and significant MR and/or significant AR and a thickened MV and/or elbow deformity of the anterior mitral leaflet
- Probable RHD: Cardiac murmur and either significant MR and(or AR or thickened MV and/or elbow deformity of the anterior mitral leaflet
- Possible RHD: No cardiac murmur and thickened mitral valve and/or elbow deformity of mitral valve and/or significant MR and/or AR
-

The documentation of a wide array of baseline variables will give us a more accurate understanding of the population at risk and help us to identify determinants of compliance to secondary prevention and eventually disease progression. We hypothesize, that lower education of the parents, lower economic status, poorer housing and overcrowding are associated with a higher prevalence of RHD.

For the cohort study, the precise documentation of clinical findings and echocardiographic lesions according to the criteria provided by the World Heart Federation at baseline and during follow-up will allow us to investigate the importance of age and stage of disease at presentation, risk factors of disease progression, and efficacy of secondary prevention for subclinical RHD. We hypothesize, that both older age at diagnosis and more advanced stage of disease at presentation are correlated with more rapid disease progression and adverse clinical outcome.

Type of Study (Specify):

Cross-sectional Survey (Part 1); Longitudinal Cohort Study (Part 2)

Cross-Sectional Survey, Cluster Randomized Trial (Part 1)

We will perform clinical and echocardiographic screening of children aged 5-15 years from public and private schools in urban and rural areas in Eastern Nepal. A multistage sampling procedure will be used to select the study sample. In order to ensure a representative target population, the location and administration of the schools will be used as a surrogate to reflect the socio-economic demographic distribution of the population in Eastern Nepal.

All schools in the Sunsari district are categorized in one out of four groups: 1. rural governmental 2. rural non-governmental 3. urban non-governmental 4. urban governmental. All schools within one group are sorted according to a randomly assigned individual number (e.g. 1-503) using research randomizer (http://www.randomizer.org/). Based on the randomly assigned increasing numbers there will be a list of schools in each group. Starting from the top of the list, 12 schools from the 4 groups will be selected in a 6:3:2:1 proportion. All children from one of those 12 selected schools (A1-A12) will then be screened for RHD at timepoint A. The next 12 schools on the randomly created lists will then be selected as the control group (A13-A24). After three years (timepoint B), all 24 schools will be revisited and all children will be screened by echocardiography. Using this approach, at timepoint B burden of morbidity among children aged 8-15 in schools A1-A12 can be compared to burden of RHD morbidity among children aged 8-15 in schools A13-A24 at timepoint B. Children younger than 8 years of age will not qualify for inclusion into this comparison, since they were not potential candidates for screening at timepoint A.

Prospective Cohort Study (Part 2)

All children with documented history of ARF and/or echocardiographic evidence of RHD will be re-examined in regular time intervals, at six months, at one year, and yearly thereafter up to at least 5 years, in the context of a prospective cohort study. Both children and their primary caregivers will be educated in order to ensure compliance with secondary prevention and regular follow-up. A standardized questionnaire will address clinical symptoms, compliance to treatment, and assess pre-specified clinical endpoints. Echocardiographic follow-up will be performed yearly up to five years at B.P. Koirala

Institute of Health Sciences (BPKIHS).

Study Site and Its Justification:

The prevalence rate of RHD is high throughout Nepal. The Sunsari district situated on the foothills of the Lower Himalayan Range in Eastern Nepal involves 52 villages with a total population of around 630,000 inhabitants. Dharan is the largest city in the Sunsari district and the third largest city in the country. BPKIHS is one of the large teaching hospitals in Nepal and will be the center of reference for echocardiographic confirmation and echocardiographic follow-up of the schoolchildren.

Study Population (Specify):

For the cross-sectional survey, all parents of the schoolchildren will be informed by a letter distributed to the children outlining the project details and indicating a contact address for queries. Since close to half of the adult population in Nepal is illiterate, focus group discussions with the health care providers, school principals, local health care workers and parents will be offered to understand and establish initiatives to win the confidence of the communities. A written informed consent form of the principal of each of the selected schools will be obtained. Schoolchildren of parents that do not actively withdraw consent for screening will be examined. Inclusion criteria for the observational survey will be as follows: age 5-15 years (1), written informed consent for participation in the screening study by the school principal (2), and passive consent from the parent/primary caregiver of the children (3). Given the observational design of the study no formal exclusion criteria apply. Children will be enrolled in the prospective registry in the presence of a documented history of ARF or echocardiographic evidence of definite or borderline RHD, and written informed consent given by the children and/or their parents/primary caregivers.

Study Unit:

Governmental and nongovernmental schools in urban and rural areas of the Sunsari district

Sampling Methods / Techniques (Specify):

According to the most recent data provided by the United Nations from the year 2011, 83% of the population of Nepal lives in rural areas (Source: http://data.un.org/CountryProfile).

Among a total of 595 schools in the Sunsari district, 503 (85%) are located in rural areas whereas 92 (15%) are located in urban areas. In addition, 383 (64%) out of the 595 schools are governmental schools, and 122 (36%) are private schools. In order to reflect this distribution we will select schools in a rural to urban ratio of 3:1, and a governmental to private ratio of 2:1. Based on these proportions, we come to a lowest common denominator of 12 schools. The methodology of cluster randomization has been outlined above under "Type of study".

Sample size (with justification):

Sample size calculations were based on reported prevalence rates of RHD using cardiac auscultation in schoolchildren in Southeast Asian countries. [1-8] We calculated a sample size of 9500 schoolchildren between the ages of 5 to 15 years with a type I error of 0.05 and a power of 90% for an expected diagnosis rate of 2 per 1000. The lower end of the prevalence estimates was chosen in order to obtain a sufficient sample size in the subsequent cohort study, and to be able to provide optimal treatment to a maximum number of affected children. We will include one urban and three rural areas from the different parts of the target area (Sunsari district), thus including 2,500 participants in each area. One third of the patients from the urban area will be enrolled from private schools.

1. Marijon E, Ou P, Celermajer DS, et al. Prevalence of rheumatic heart disease deteceted by echocardiographic screening. N Engl J Med 2007;357:470-6.
2. Bhaya M, Panwar S, Beniwal R, et al. High prevalence of rheumatic heart disease detected by echocardiography in school children. Echocardiography 2009;27:448-453.
3. Saxena A, Ramakrishnan S, Roy A, et al. Prevalence and outcome of subclinical rheumatic heart disease in India: The RHEUMATIC (Rheumatic Heart Echo Utilization and Monitoring Actuarial Trends in Indian Children) study. Heart 2011;97:2018-2022.
4. Sadiq M, Islam K, Abid R, et al. Prevalence of rheumatic heart disease in school children in urban Lahore. Heart 2009;95:353-357.
5. Bahadur KC, Sharma D, Shrestha MP, et al. Prevalence of rheumatic and congenital heart disease in schoolchildren of Kathmandu valley in Nepal. Indian Heart J 2003;5(6):615-8.
6. Jose VJ, Gomathi M. Declining prevalence of rheumatic heart disease in rural schoolchildren in India: 2001-2002. Indian Heart J 2003;55(2):158-60.
7. Ahmed J, Mostafa Zaman M, Monzur Hassan MM. Prevalence of rheumatic fever and rheumatic heart disease in rural Bangladesh. Trop Doct 2005;35(3):160-1.
8. Periwal KL, Gupta BK, Panwar RB, et al. Prevalence of rheumatic heart disease in school children in Bikaner: an echocardiographic study. J Assoc Physicians India 2006;54:279-82.

Data Collection Technique / Methods (Specify):

Data acquisition will be organized in a three-staged process by study nurses and physicians. Data on social background and past medical history will be acquired in a standardized interview on the basis of a questionnaire. Demographic variables such as age, household characteristics, and socio-economic indicators will be recorded along with a short medical history followed by physical examination documenting height, weight, and potential clinical signs of ARF. Study nurses will question the children about demographic characteristics, fill in the questionnaire, and measure height and weight. A first physician will complete the medical history and execute physical examination including cardiac auscultation. A second independent physician will perform screening echocardiography to document morphologic and/or functional valvular lesions consistent with RHD. All children with valvular lesions consistent with RHD will be included into a prospective registry. The parents or caregivers will be contacted and invited to BPKIHS outpatient clinic for detailed information on RHD and education on the importance of secondary prevention. Secondary antibiotic prevention will be provided free of charge every 3-4 weeks at BPKIHS. After informed consent, clinical and echocardiographic follow-up will be organized on a regular basis after 6 months, after 1 year and yearly thereafter until 5 years. Parents of the children will be reminded of the follow-up visits by telephone calls.

Data Collection Tools: (please attached in annex)

Case Report Forms for the Observational Survey and the longitudinal cohort study will be provided in the annex.

Pre-testing the Data Collection Tools (if applicable):

A pilot study has been performed in order to test the data collection tools:

Objectives: To evaluate a protocol for a population-based program targeting prevention of rheumatic heart disease (RHD) progression by early echocardiographic diagnosis of valvular lesions and timely implementation of secondary prevention.

Design: Observational survey with a subsequent prospective cohort study.

Setting: Private boarding school in the urban area of the Sunsari district situated on the foothills of the Lower Himalayan Range in Eastern Nepal.

Participants: Fifty-four unselected school going children 5-15 years of age, 24 girls and 30 boys.

Primary outcome measure: Logistic feasibility of a large-scale population-based screening study using the echocardiographic criteria formulated by the World Heart Federation, with longitudinal follow-up of children with definite or borderline RHD in a prospective cohort study.

Results: Standardized interview, physical examination and screening echocardiography were performed in a three-staged process and took approximately 6 minutes per child. Socio-economic status was assessed using surrogate markers such as the occupation of the primary caregiver, numbers of rooms at home, car, television, cell phone and internet connection. Physical examination was focused on cardiac auscultation and signs of acute rheumatic fever and targeted echocardiography was performed by an independent examiner without knowledge of the clinical findings. Two children with evidence of borderline RHD were re-examined at B.P. Koirala institute of Health Sciences and the indication for secondary antibiotic prevention was discussed with the parents and the children. At six months of follow-up, echocardiographic findings were stable in both children. Implementation of secondary antibiotic prevention was challenged by impaired awareness of subclinical RHD among parents and inadequate cooperation with family physicians.

Conclusions: This pilot study shows that the methods outlined in the protocol can be translated into a large-scale population-based study. We learned that education and collaboration with teachers, parents and family physicians/pediatricians will be of key importance in order to establish a sustainable program.

Trial registration: ClinicalTrials.gov Identifier: NCT01550068

Shrestha NR, Kalesan B, Karki P, Sherpa K, Basnet A, Urban P, Pilgrim T. Rheumatic heart disease: pilot study for a population-based evaluation of prevalence and cardiovascular outcomes among schoolchildren in Nepal. BMJ Open. 2012;2(5).

Validity and Reliability of the Study Tools:

Echocardiography is the gold standard to detect clinically silent RHD. Criteria for diagnosis of RHD have been recently published by the World Heart Federation:

Echocardiographic diagnosis will classify RHD according to the WHF criteria for individuals aged ≤20 years into definite and borderline. Definite RHD is further subdivided into four subcategories. Subcategory A is pathological mitral regurgitation and at least two morphological features of RHD of the mitral valve, subcategory B is the presence of mitral stenosis with a mean gradient of ≥4 mmHg. Subcategory C is defined by pathological aortic regurgitation in combination with at least two morphological features of RHD of the aortic valve, and subcategory D is determined by borderline disease of both the aortic valve and the mitral valve. Borderline RHD is subdivided into three subcategories. Subcategory A is the presence of at least two morphological features of RHD of the mitral valve without pathological mitral regurgitation or mitral stenosis, subcategories B and C are determined by pathological mitral regurgitation, or pathological aortic regurgitation, respectively. Physiological mitral regurgitation (A), physiological aortic regurgitation (B), and an isolated morphological feature of RHD of the mitral or aortic valve (i.e. valvular thickening) without any associated pathological stenosis or regurgitation (C and D, respectively) will be classified as normal echocardiographic findings.

Potential Biases (if applicable):

Even though the population attributable risk of RHD is expected to be high, false positive screening results may occur. In order to reduce false positive findings all echocardiographic clips with borderline or definite RHD will be assessed from two independent cardiologists. Moreover, screening in a large population of schoolchildren might yield in exceptional cases important incidental clinical findings unrelated to RHD but yet relevant for future prognosis (i.e. bicuspid aortic valve, atrial septal defect). Parents will be informed about such findings and advised on how to proceed for best medical management. If the parents or care-takers give their consent, the children will be invited for follow-up at BPKIHS for further work-up. First medical contact due to incidental findings relevant for future prognosis will be reimbursed.

Limitation of the Study:

Since access to education is a marker of socio-economic status, restriction of screening to school-going children is subjected to selection bias likely to underestimate the real disease burden related to RHD in Eastern Nepal.

16. Plan for Supervision and Monitoring:

The study will be centrally monitored by the clinical trials unit at University of Bern, Switzerland.

17. Plan for Data Management and Analysis:

In the cross-sectional study, age and sex specific prevalence rates for RHD will be calculated by socio-demographic covariates. The design effect of the cluster sampling strategy will then be calculated using the variations of prevalence rates among the different clusters. A multiplication factor equal to the square root of the design effect will be used to construct the 95% confidence intervals (CIs) for the prevalence estimates to avoid erroneously narrow confidence intervals (CI). Prevalence rates with 95% CI will be calculated separately for the two screening methods. Multivariable analysis will be performed for assessing the socio-economic factors for RHD. Among children with RHD, the socio-economic barriers to receive adequate medical care will be assessed by using a multivariate logistic regression model. Furthermore, the association of RHD with age, gender, socio-economic status, and urban–rural residence will be evaluated with univariate and multivariate analyses.

Cohort baseline characteristics and procedural variables will be presented as counts and percentages for dichotomous variables and as mean and standard deviation (SD) for continuous variables. Factor analysis of the scales in QOL will be performed using appropriate rotations and index scores of the constructs will be computed if not more than 25% of the items are missing. Adherence to treatment will be measured as a dichotomous variable and quality of life (QOL) scores as mean (SD) / median (interquartile range) will be presented for each follow up time interval. We will present and compare the baseline and procedural characteristics by the stages of disease progression at baseline using chi-square tests or analyses of variance. Comparisons of the baseline characteristics of the study subjects among the disease stages at baseline will be performed using linear or logistic regression. For the specific clinical endpoints, compliance and QOL univariable and multivariable Cox proportional hazard regression models will be used to calculate hazard ratios with 95% CI among the stages of disease at baseline. We will construct Kaplan–Meier curves for the time to the development of clinical endpoints by stage of disease at baseline, treating death as a competing risk.

18.Expected Outcome of the Research:

More than 80% of the children younger than 15 years of age grow up in regions of the world where rheumatic heart disease is endemic. The results of the present study may have a far-reaching impact on health resource utilization in developing and emerging countries with endemic rheumatic heart disease where it causes 250,000 deaths every year and affects more than 15 million people. The relatively novel entity of clinically silent rheumatic heart disease introduced by the increased sensitivity of echocardiography screening challenges our current concept of secondary prevention. Diagnosis of early stages of valvular lesions due to rheumatic heart disease in school going children and timely implementation of secondary antibiotic prevention may prevent disease progression leading to morbidity and mortality.

19.Plan for Dissemination of Research Results:

The study protocol has been was registered with 'www.ClinicalTrials.gov' (NCT01550068) and was published in BMJ Open (Pilgrim T, Kalesan B, Karki P, et al. Protocol for a population-based study of rheumatic heart disease prevalence and cardiovascular outcomes among schoolchildren in Nepal. BMJ Open 2012;2(3). pii: e001320.) The study findings will be reported in peer-reviewed publications.

20.Plan for Utilization of the Research Findings (optional):

The study addresses a subject of global dimension. More than 80% of the children younger than 15 years of age grow up in regions of the world where rheumatic heart disease is endemic. The results of the present study have an important impact on health resource utilization in Nepal.

The relatively novel entity of clinically silent rheumatic heart disease introduced by the increased sensitivity of echocardiography screening challenges our current concept of secondary prevention. If the study demonstrates that secondary prevention for silent rheumatic heart disease minimizes progression of disease to adverse clinical outcome, this finding will reinforce the call for routine echocardiographic screening for rheumatic heart disease and require re-consideration of the current guidelines for secondary antibiotic prevention.

How is the research project going to strengthen the research capability of the host institution: Nepali Researcher (if submitted from abroad):

The scientific exchange will serve as a platform for mutual learning. We acknowledge that capacity-strengthening will be of key importance throughout the course of the study and are making considerable efforts to guarantee sustainability of the project. Capacity-strengthening will occur on different levels. First, selected fellows from BPKIHS involved in the screening process will be trained in echocardiography using portable ultrasound devices. Second, the Department of Cardiology at the University of Bern will provide clinical proctoring for invasive treatment strategies required for children identified with cardiac disease during screening (mitral balloon valvuloplasty, ASD closure). Dr Nikesh Shrestha has already completed an interventional cardiology fellowship program at Bern University between January and September 2010 which formed the basis for a continued exchange between the two institutions. This transfer of knowledge will be the basis for a sustainable program. Third, study personnel will be educated on good clinical practice (GCP) and instructed on database management and statistical analysis.

We will be open to enroll an interested fellow for higher degrees and will be happy to involve local students and residents to perform sub-analyses of the database and will assist in providing support where needed.

As an institution, BPKISH will be involved in an international network of rheumatic heart disease research and closely collaborate with the Clinical Trials Unit at Bern University Hospital in Switzerland.

21. Work Plan *(should include duration of study, tentative date of starting the project and work schedule / Gantt chart):*

Date	Milestone	Responsibility
Approval Institutional review board, ethics committee;	February 2012	Cardiology, Bern<br>Cardiology, BPKIHS

registration with www.clinicaltrials.gov		
Publication of study protocol	May 2012	
Pilot Study for phase I and phase II	February to August 2012	Cardiology, Bern<br>Cardiology, BPKIHS
Publication of pilot study	September 2012	
Cluster randomization of schools, submission to district education office	September 2012	Cardiology, Bern<br>Cardiology, BPKIHS
Orientation of school principals	September 2012	Cardiology, BPKIHS
Orientation of parents about observational survey	September 2012	Cardiology, Bern<br>Cardiology, BPKIHS
Installation of REDCap electronic data capture tools	September 2012	ISPM, Bern
Cross-sectional survey	January 2013 to January 2014	Cardiology, BPKIHS
Publication of cross-sectional survey	April 2014	
Longitudinal cohort study	2013-2019	Cardiology, BPKIHS
Mathematical modelling	2019	ISPM, Bern
*Publication of mathematical mo*del	2019	

Part – IV

Ethical Consideration

22. Regarding the human participants:

Are human participants required in this research? If yes, provide justification.

[x] Yes (*provide justification*) No [ ]

Screening echocardiography will be performed among school going children 5 to 15 years of age since the occurrence of rheumatic fever ist highest in this age group and early detection is expected to have to greatest benefit on future prognosis. Screening echocardiography is a non-invasive examination without radiation exposure that is not associated with any harmful complications.

How many participants are required for the research? Explain.

We calculated a sample size of 9500 schoolchildren between the ages of 5 to 15 years with a type I error of 0.05 and a power of 90% for an expected diagnosis rate of 2 per 1000.

What is the frequency of the participant's involvement in the research? Explain.

All children with documented history of ARF and/or echocardiographic evidence of RHD will be re-examined in regular time intervals, at six months, at one year, and yearly thereafter up to at least 5 years, in the context of a prospective cohort study. Both children and their primary caregivers will be educated in order to ensure compliance with secondary prevention and regular follow-up. A standardized questionnaire will address clinical symptoms, compliance to treatment, and assess pre-specified clinical endpoints. Echocardiographic follow-up will be performed yearly up to five years at B.P. Koirala Institute of Health Sciences (BPKIHS).

Clearly indicate the participant's responsibilities in the research. What is expected of the research participants during the research?

We anticipate that compliance with secondary prevention will be one of the major challenges, which in turn requires continued education. We plan to ensure compliance by means of face-to-face education by the research coordinator at each visit to both the child and the caregiver, making them aware of the risk of recurrence of rheumatic fever and the potential consequences of progression of rheumatic heart disease. Visit reminders by mail and if necessary by personal visits will also be used to ensure adequate compliance.

Are vulnerable members of the population required for this research? If yes, provide justification.

Screenng examinations will be performed in schoolchildren since the prevalence of silent rheumatic heart disease and therefore the burden of still preventable morbidity and mortality is greatest among children 5-15 years of age.

Are there any risks involved for the participants? If yes, identify clearly what are the expected risks for the human participants in the research and provide a justification for these risks.

Primary caregivers will be educated to detect symptoms and signs of potential allergic reactions of antibiotic treatment such as a skin rash, hives, swollen lips/tongue and wheezing , and will be provided with an emergency medical contact number. In case of drug-related adverse side effects such as diarrhea, nausea, pain/swelling/infection at the site of injection, we will recommend to switch the antibiotic administration from intramuscular to oral or vice versa as a first step. If children/caregivers refuse regular antibiotic intake despite counseling, they will have the option to retract their decision at any given point in time. With their consent, these children will remain in the registry in order to provide care and treatment in case of potential late-complications as a consequence of disease progression. In the event of children being

diagnosed at an advanced stage with significant valvular heart disease leading to congestive heart failure, we will assure optimal interdisciplinary medical treatment.

Are there any benefits involved for the participants? If yes, identify clearly what are the expected benefits for the participants.

Early implementation of secondary antibiotic prevention my prevent progression of rheumatic heart disease.

23. Informed Consent Form / Ethical Issues:

Statements required in the Informed Consent Form include:

A statement that the human participants can withdraw from the study at any time without giving reason and without fear. State clearly how the participants can opt out the study.

A statement guaranteeing the confidentiality of the research participants.

If required, a statement on any compensation that might be given to the research participant and or their community.

A statement indicating that the participants has understood all the information in the consent form and is willing to volunteer / participate in the research.

Signature space for the research participants, a witness, and the date.

(Informed Consent form should be submitted in English and in the language appropriate to the research participants)

Obtaining the Consent
How informed consent is obtained from the research participants?

[ ] Verbal [x] Written

Please indicate who is responsible for obtaining informed consent from the participants in this research study?

The local study coordinator will collect the consent from the parents of the children.

Is there anything being withheld from the research participants at the time the informed consent is being sought?

No, there is nothing withheld from the research participants.

If yes, explain

....

Is the research sensitive to the Nepali culture and the social values?

Yes [✓] No [ ] Explain.

....

Is health insurance *(if applicable)* being made available to the research participants? If yes, please provide the necessary insurance data.

There is no particular health insurance available to the research participants, however, secondary antibiotic prevention as well as follow-up examinations will be for free for the study participants.

(Include in consent form)

24. Regarding Clinical Trial:

The study is not a clinical trial, but a cross-sectional survey with a subsequent registry of patients with rheumatic heart disease.

In case of a clinical trial address the following:
The trial treatment

....

A detailed explanation of the trial procedures including all invasive procedures.

....

The potential or direct benefits (if any) for the research participants.

....

Alternative procedure(s) or treatment(s) that may be available.

....

The risks, discomforts, and inconveniences associated with the study

....

Provisions for management of any adverse reactions

....

The provisions of insurance coverage for any permanent disability or death caused directly by the investigational treatment or procedure.

....

The provision of including the name and address, including telephone numbers of person to be contacted in case of adverse events or for any information related to the trial.

....

Is there going to be a transfer of any biological materials from the country? Explain.

....

Is there a Data Safety Monitoring Board?
If Yes, Mention

....

Is this trail internationally registered?

Yes, the trial was registered with 'www.ClinicalTrials.gov' (NCT01550068)..

Part – V

ACCEPTANCE OF GENERAL CONDITIONS AND DECLARATION BY THE PRINCIPAL INVESTIGATOR

I hereby certify that the above mentioned statements are true, I have read and understood the regulation of the Nepal Health Research Council (NHRC) on the approval of research proposal and will act in conformity with the said regulation in all respects.

If the research is terminated, for any reason, I will notify NHRC of this decision and provide the reasons for such actions. I will provide NHRC with a written notice upon the completion of the research as well as a final summary/full report of the research study. If I publish the results in a journal, I shall acknowledge the NHRC and shall provide the Council with three copies of any such articles.

....

Signature of Applicant **Date:** ....

INFORMED CONSENT:

- Describe the manner in which informed consent will be obtained.
- Indicate what kind of consent (e.g. parental, child, adult, etc) will be used.
- If the subjects are children/adolescents ages 7-18 years, an Assent Form must be included with the IRB application. The signed Assent Form along with the Parental/Guardian Consent Form must be retained on file for at least three years after completion of the research project.
- If prisoners / pregnant women, or fetuses are to be included in the research sample, it is likely that a full IRB review will be required and additional human subjects' protections will be expected.
- If the subjects do not read or comprehend English, you must provide a consent form in their language as well as in English for IRB review and approval.
- If you are requesting a waiver of written consent (i.e. a signature on an informed consent form) from the subjects, you MUST justify this request by providing an explanation of why obtaining written consent would add additional risk to the subjects and your alternative provisions for informing them about the study.
- If consent documents from another site will be used, you will have to indicate this and provide a copy of the authorized consent document and IRB approval with your application.
- You will have to provide any other relevant information if necessary. Please be aware that the PI is legally required to retain all signed Informed Consent forms for at least three years after the project terminates
- The Informed Consent form must be written at a level that the subjects will understand. Please use simple language, and avoid clinical jargon.
- Attach a copy of the written informed consent form (assent or parental consent where applicable). Consent documents MUST be in format requested. See examples on line.
- If the study uses database or archival data the use of informed consent is not applicable.

CONFIDENTIALITY OF DATA: *Confidentiality of data MUST be address for all studies.*

- Indicate the extent to which confidentiality of records identifying subjects will be maintained.
- Describe the storage and disposal of information where applicable.

Check List

For all applicants

1. Covering letter addressed to the Member secretary indicating the submission of the approval of proposal.
2. Proposal will only be accepted if submitted in NHRC format.
3. Both printed and electronic version of the proposal should be submitted.
4. Curriculum Vitae of the Principal Investigator & Co-Principal Investigator of the study team should be submitted.
5. If the Principal Investigator is a non Nepali citizen, at least one Co-investigator should be a Nepali citizen.
6. Submission of the application processing fee to NHRC.(According to NHRC rules and regulations)
7. Source of funding for the proposed project.
8. The proposal should have institutional ethical clearance from his/her own country if submitted from academic and related institution.
9. If the research study is to be conducted in any hospitals/organization or institution/community, a letter of approval from the related hospital/organization or institution/district authority should be provided.
10. Consent form should be in Nepali & local language (if necessary).
11. Data collection tools should be in Nepali & local language (if necessary) including interview guideline, observation checklist, questionnaires etc.
12. Style of referencing should be in Harvard style.
13. List of abbreviations / acronyms should be provided.

For students' applicants

1. Approval letter from concern Institute/University.
2. Recommendation letter from Academic Supervisor.

Processing Fee

Researcher has to pay the processing fee as per the rules and regulations of NHRC.

Nepal Health Research Council
Estd. 1991

NHRC

Ref. No. 273

Executive Committee

Executive Chairman
Prof. Dr. Chop Lal Bhusal

Vice - Chairman
Dr. Rishi Ram Koirala

Member-Secretary
Dr. Shanker Pratap Singh

Members
Prof. Dr. Meeta Singh
Prof. Dr. Suman Rijal
Dr. Narendra Kumar Singh
Dr. Samjhana Dhakal
Dr. Devi Gurung

Representative
Ministry of Finance
National Planning Commission
Ministry of Health & Population
Chief, Research Committee, IOM
Chairman, Nepal Medical Council

1 September2013

Dr. Thomas Michael Pilgrim
Principal Investigator
Swiss Cardiovascular Center,
Bern University Hospital, Switzerland

Ref: **Approval of Research Proposal** entitled **Population – based study of rheumatic heart disease prevalence and cardiovascular outcomes of secondary prevention among schoolchildren in selected district of Eastern Nepal**

Dear Dr. Pilgrim,

It is my pleasure to inform you that the above-mentioned proposal submitted on 2 August 2013 (**Reg. no. 109/2013** please use this Reg. No. during further correspondence) has been approved by NHRC Ethical Review Board on 29 August 2013.

As per NHRC rules and regulations, the investigator has to strictly follow the protocol stipulated in the proposal. Any change in objective(s), problem statement, research question or hypothesis, methodology, implementation procedure, data management and budget that may be necessary in course of the implementation of the research proposal can only be made so and implemented after prior approval from this council. Thus, it is compulsory to submit the detail of such changes intended or desired with justification prior to actual change in the protocol.

If the researcher requires transfer of the bio samples to other countries, the investigator should apply to the NHRC for the permission.

Further, the researchers are directed to strictly abide by the National Ethical Guidelines published by NHRC during the implementation of their research proposal and submit progress report and full or summary report upon completion.

As per your research proposal, the total research amount is US$. 9,885.00 and accordingly the processing fee amounts to NRs. 9,710.00. It is acknowledged that the above-mentioned processing fee has been received at NHRC.

If you have any questions, please contact the research section of NHRC.

Thanking you.

....
Dr. Shanker Pratap Singh
Member Secretary

Tel.+977-1-4254220, 4227460, Fax: +977-1-4262469, RamShah Path, P.O. Box 7626, Kathmandu, Nepal.
