# Supplementary material for: Effectiveness of Systematic Echocardiographic Screening for Rheumatic Heart Disease in Nepalese Schoolchildren: A Cluster Randomized Clinical Trial
Source: JAMA Cardiol. 2021 Jan 20;6(4):1–7. doi: 10.1001/jamacardio.2020.7050 (PMC7818193; doi:10.1001/jamacardio.2020.7050)
Supplement: Supplement 2. — eMethods. Supplemental Methods eTable. Estimated Costs for Screening of All Schools in the Sunsari District eFigure 1. Timeline of Echocardiographic Follow-up in Experimental Schools (Red) and Control Schools (Blue) eFigure 2. Spaghetti Plot Displaying Longitudinal Data of Children in the Experimental Arm With Definite Rheumatic Heart Disease Who Were in the Ineligible Age Range for Inclusion Into the Cluster Randomized Comparison (Age >12 Years at Baseline) and Underwent Echocardiographic Follow-up Outside the Auxiliary Cross-sectional Screening eReferences [file jamacardiol-e207050-s002.pdf]

Supplementary Online Content

Karki P, Uranw S, Bastola S, et al. Effectiveness of systematic echocardiographic screening for rheumatic heart disease in Nepalese schoolchildren. *JAMA Cardiol.* Published online January 20, 2021. doi:10.1001/jamacardio.2020.7050

eMethods. Supplemental Methods

eTable. Estimated Costs for Screening of All Schools in the Sunsari District

eFigure 1. Timeline of Echocardiographic Follow-up in Experimental Schools (Red) and Control Schools (Blue)

eFigure 2. Spaghetti Plot Displaying Longitudinal Data of Children in the Experimental Arm With Definite Rheumatic Heart Disease Who Were in the Ineligible Age Range for Inclusion Into the Cluster Randomized Comparison (Age >12 Years at Baseline) and Underwent Echocardiographic Follow-up Outside the Auxiliary Cross-sectional Screening

eReferences

This supplementary material has been provided by the authors to give readers additional information about their work.

© 2021 American Medical Association. All rights reserved.

eMethods. Supplemental Methods

Data collection

Echocardiographic examinations were coordinated by the B.P. Koirala Institute of Health Sciences and performed by trained physicians familiar with the local context. The protocol for data collection was described previously.[1,2] Data were collected using the REDCap electronic data capturing system hosted and centrally monitored by the Clinical Trials Unit of the University of Bern.[3] Data from screening examination and follow-up in experimental schools were therefore linked using Western translations of Nepalese names, and Western dates of birth based on the Gregorian calendar as calculated from Nepalese dates of birth. Neither Nepalese names nor dates of birth of children were directly available from school administrations and were therefore reported by children themselves. The number of children available at follow-up and the proportion of children that could be expected to be linked in experimental schools was predicted from age-specific out-of-school statistics reported separately for urban and rural areas of the district of Sunsari.[4] Errors in children-reported Nepalese names and dates of birth, variations in Western translations of names, and the unreliability of reported age and dates of birth in Nepal where only 56% of children under the age of 5 are found to have their births registered[5] resulted in a considerably lower success of linkage between baseline and follow-up of children in experimental schools than what we anticipated based on out-of-school statistics.

Projection for the implementation of school-based screening program in the entire Sunsari district

To project volume, workload, cost and yield of screening if a school-based systematic screening program were introduced in the entire Sunsari district, we assumed that 3 teams would screen approximately 300,000 children aged ≥8 years during a 10-year period. We calculated the cumulative cost of salaries, equipment, vehicles (including amortisation), and gas in US $ and estimated the yield of children with definite or borderline RHD by applying the prevalence estimate and 95% CI from control schools of our study to the projected number of children screened during 10 years in the Sunsari district.

© 2021 American Medical Association. All rights reserved.

eTable. Estimated Costs for Screening of All Schools in the Sunsari District

**Staff costs**		Amount (USD)	Number per team
	Physician	10,560	2
	Nurse	2,640	2
	Driver	2,000	1
	Cost per team	28,400	
**Equipment cost**			Years of amortization
	Portable echocardiography machine	25,000	5
	Power generator	1,000	10
**Transportation cost**			
	Minibus	15,000	10
	Fuel costs per year	1,000	
	Repair costs per year, general	1000	
	Cost per year per team	18,500	
Number of years		*10*	
Number of teams		*3*	
Cost per year for all 3 teams			
	Staffing cost per year	85,200	
	Infrastructure cost per year	55,500	
	Subtotal	140,700	
**Estimated total cost for full study duration**			
		**1,407,000**	

© 2021 American Medical Association. All rights reserved.

eFigure 1. Timeline of Echocardiographic Follow-up in Experimental Schools (Red) and Control Schools (Blue)
The x-axis indicates time, the y-axis on the left side indicates the number of children (blocks), the y-axis on the right side the number of schools (lines).

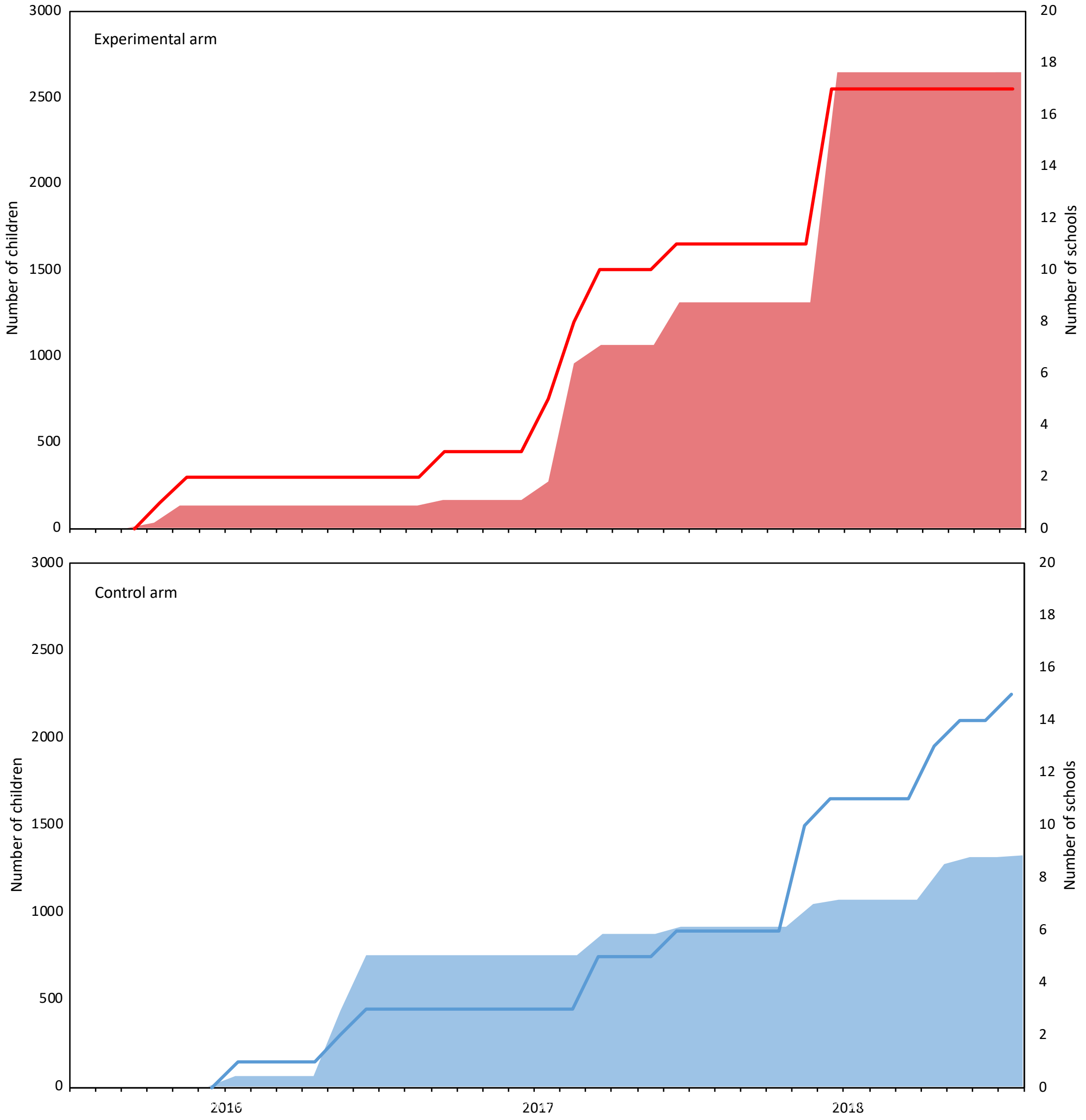

© 2021 American Medical Association. All rights reserved.

eFigure 2. Spaghetti Plot Displaying Longitudinal Data of Children in the Experimental Arm With Definite Rheumatic Heart Disease Who Were in the Ineligible Age Range for Inclusion Into the Cluster Randomized Comparison (Age >12 Years at Baseline) and Underwent Echocardiographic Follow-up Outside the Additional Cross-sectional Screening

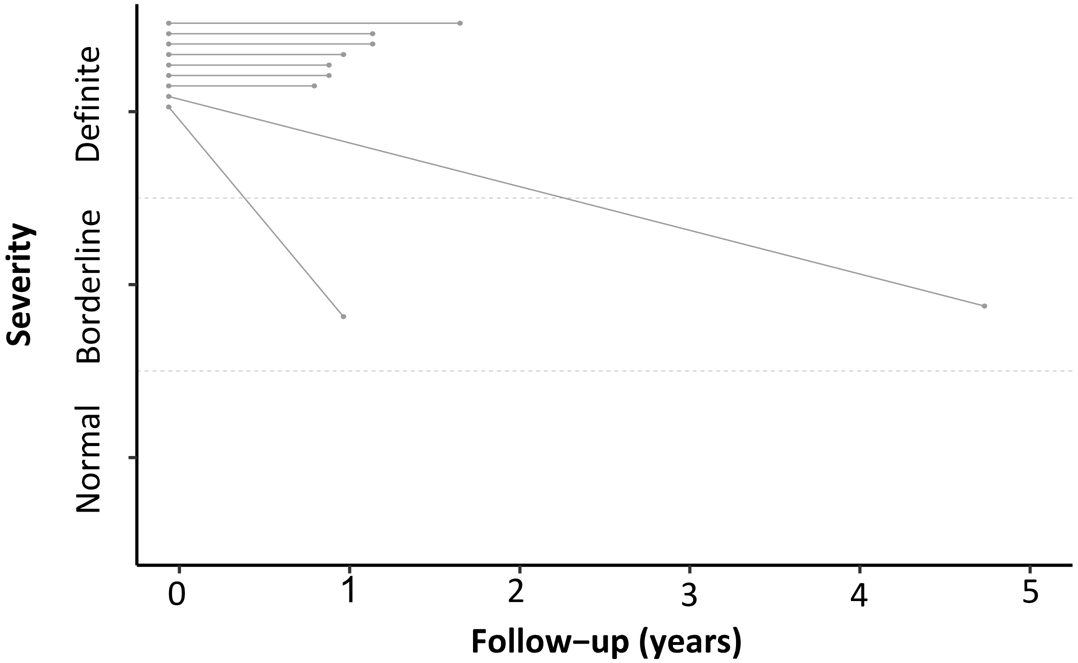

© 2021 American Medical Association. All rights reserved.

eReferences

1. Shrestha NR, Karki P, Mahto R, et al. Prevalence of Subclinical Rheumatic Heart Disease in Eastern Nepal: A School-Based Cross-sectional Study. *JAMA Cardiol*. 2016;1(1):89-96.

2. Pilgrim T, Kalesan B, Karki P, et al. Protocol for a population-based study of rheumatic heart disease prevalence and cardiovascular outcomes among schoolchildren in Nepal. *BMJ Open*. 2012;2(3).

3. Harris PA, Taylor R, Thielke R, Payne J, Gonzalez N, Conde JG. Research electronic data capture (REDCap)--a metadata-driven methodology and workflow process for providing translational research informatics support. *J Biomed Inform*. 2009;42(2):377-381.

4. Ministry of Education, United Nations Children's Fund (UNICEF) and United Nations Educational, Scientific and Cultural Organization (UNESCO), Global Initiative on Out of School Children - Nepal Country Studiy, July 2016, UNICEF, Kathmandu, Nepal, 2016.

5. UNICEF global databases, 2019, http://data.unicef.org/topic/child-protection/birth-registration/ accessed on February 27, 2019.

© 2021 American Medical Association. All rights reserved.
