# Supplementary material for: Effectiveness of Systematic Echocardiographic Screening for Rheumatic Heart Disease in Nepalese Schoolchildren: A Cluster Randomized Clinical Trial
Source: JAMA Cardiol. 2021 Jan 20;6(4):1–7. doi: 10.1001/jamacardio.2020.7050 (PMC7818193; doi:10.1001/jamacardio.2020.7050)
Supplement: Supplement 3. — Data sharing statement [file jamacardiol-e207050-s003.pdf]

Data Sharing Statement

Karki. Effectiveness of Systematic Echocardiographic Screening for Rheumatic Heart Disease in Nepalese Schoolchildren. *JAMA Cardiol*. Published January 20, 2021. doi:10.1001/jamacardio.2020.7050

Data
Data available: Yes
Data types: Deidentified participant data
How to access data: www.rhedproject.org
When available: With publication

Supporting Documents
Document types: Statistical/analytic code
How to access documents: www.rhedproject.org
When available: With publication

Additional Information
Who can access the data: The data will be made available to anyone requesting the data.
Types of analyses: The data will be made available for any non-commercial Purpose.
Mechanisms of data availability: The data will be made available without Investigator support.
